# Understanding general practitioner and pharmacist preferences for pharmacogenetic testing in primary care: a discrete choice experiment

**DOI:** 10.1038/s41397-024-00344-z

**Published:** 2024-08-09

**Authors:** John H. McDermott, Videha Sharma, Glenda M. Beaman, Jessica Keen, William G. Newman, Paul Wilson, Katherine Payne, Stuart Wright

**Affiliations:** 1 Manchester Centre for Genomic Medicine, St Mary’s Hospital, Manchester University Hospitals NHS Foundation Trust, Manchester, M13 9WL UK; 2 Division of Evolution, Infection and Genomics, School of Biological Sciences, The University of Manchester, Manchester, UK; 3 Division of Informatics, Centre for Health Informatics, Imaging and Data Science, School of Health Sciences, The University of Manchester, Manchester, United Kingdom; 4 Centre for Primary Care and Health Services Research, Division of Population Health, Health Services Research and Primary Care, School of Health Sciences, The University of Manchester, Manchester, UK; 5 Manchester Centre for Health Economics, Division of Population Health, Health Services Research and Primary Care, School of Health Sciences, The University of Manchester, Manchester, UK

**Keywords:** Health services, Genetic markers

## Abstract

Pharmacogenetic testing in the United Kingdom’s National Health Service (NHS) has historically been reactive in nature, undertaken in the context of single gene-drug relationships in specialist settings. Using a discrete choice experiment we aimed to identify healthcare professional preferences for development of a pharmacogenetic testing service in primary care in the NHS. Respondents, representing two professions groups (general practitioners or pharmacists), completed one of two survey versions, asking them to select their preferred pharmacogenetic testing service in the context of a presentation of low mood or joint pain. Responses from 235 individuals were included. All respondents preferred pharmacogenetic testing over no testing, though preference heterogeneity was identified. Both professional groups, but especially GPs, were highly sensitive to service design, with uptake varying depending on the service offered. This study demonstrates uptake of a pharmacogenetic testing service is impacted by service design and highlights key areas which should be prioritised within future initiatives.

## Introduction

In 2022-23, there were 1.18 billion prescription items dispensed in the community in England, costing an estimated £10.4 billion, an increase of 14% over the past decade [1]. This growth has taken place over a period where the volume and complexity of contacts in primary care have also risen considerably. The NHS Long Term Plan, published in 2019, recognised this increasing demand and highlighted the importance of medicines optimisation to realise more effective use of the prescribing budget in primary care [2]. The plan committed to developing strategies to support informed prescribing practice, reducing the impact of ineffective treatment, avoiding over prescribing, and preventing problematic polypharmacy.

One emerging approach to support medicines optimisation is by selecting treatment based pharmacogenetic variation. There is good evidence that the effectiveness and safety of many commonly prescribed medicines are influenced by genetic variants which are common in the UK population [3]. Medicines such as antidepressants, antiplatelets, proton pump inhibitors, statins, and opioid analgesics all have evidence-based guidance for pharmacogenetic guided prescribing, many of which have been adopted into routine practice by other healthcare systems [4]. In England, there is a policy impetus to accelerate embedding the use of genomic medicine across the NHS [5].

Routine implementation of pharmacogenetics could improve patient outcomes, whilst directing more effective use of the healthcare budget through avoidance of therapeutic failure and adverse drug reactions (ADRs), reducing GP reattendance or admission to hospital [6, 7]. A remaining challenge, however, is how to implement pharmacogenetic guided prescribing in a complex adaptive environment where the mounting pressures are already considered unsustainable [8]. This necessitates a service to be developed which allows tests to be ordered, samples to be genotyped, and results to be returned to facilitate informed prescribing without disrupting routine practice.

In order for pharmacogenetic guided prescribing to be used routinely, the service it is embedded within must meet the needs of health care professionals (HCPs). One approach to explore preferences for a new intervention is a discrete choice experiment (DCE), a survey-based method used to elicit choices to quantify stated preferences [9]. DCEs have previously been used to assess preferences for the key attributes of a pharmacogenetic testing service within specific clinical scenarios, but not to investigate preferences in relation to a pharmacogenetic testing service for primary care in the NHS [9–13]. Via a DCE, this study aimed to identify preferences of relevant HCPs likely to be involved in the delivery of a pharmacogenetic testing service in primary care.

## Methods

**Fig. 1:**
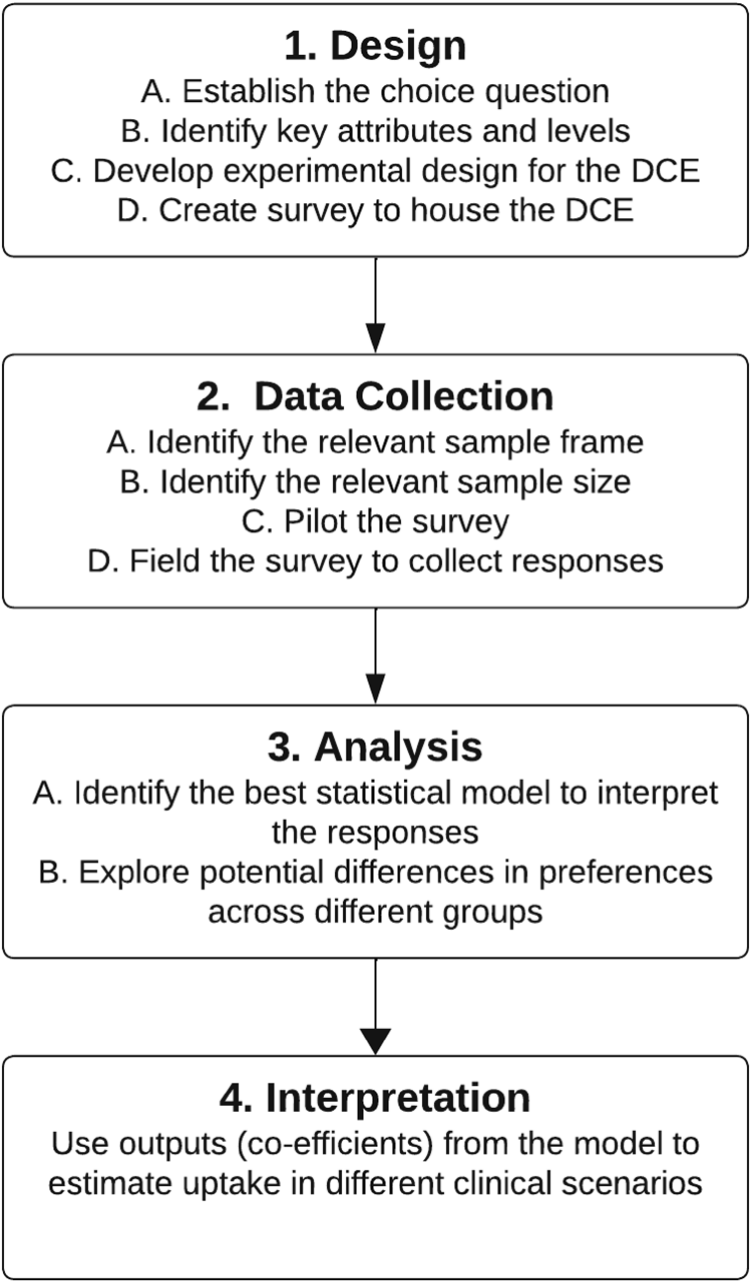
Methodological steps to perform and analyse a discrete choice experiment.

The design, application, and analysis of a DCE involve a series of four methodological steps (Fig. 1), summarised below and informed by previously published guidelines [14].

### Design

#### Establishing the choice question

The choice question was “If you had to choose one of these pharmacogenetic tests (Test A or Test B) to help guide your patient’s treatment, which would you choose?”. The DCE used an unlabelled design and included an opt-out, representing “no pharmacogenetic test” to reflect current prescribing so that potential uptake of a pharmacogenetic test in primary care could be investigated.

#### Identifying attributes & levels

Two surveys were designed in the context of two clinical scenarios in which a patient had presented in primary care and pharmacogenetic testing could be used to inform prescribing. One scenario was joint pain, and the other was low mood. There are established clinical guidelines to support pharmacogenetic informed prescribing for both these exemplar clinical scenarios [15, 16]. Attributes and levels for the DCE (Table 1) were selected and framed with support of clinical stakeholders via structured focus groups (Supplementary Appendix 1).

**Table 1:** Attributes and levels developed for the discrete choice experiment.

Attributes	Label for DCE	Levels	Type of data/coding
Type of data reported	The volume and complexity of pharmacogenetic data reported to healthcare professional	Focused - results are only provided in relation to a single medicine - i.e. the medicine for which the pharmacogenetic test was initially requested.	Categorical
		Narrow – Prescribing guidance will be returned in relation to other gene-drug pairs where strong evidence exists. Approximately 5 genes related to 20 medicines.	
		Broad - Prescribing guidance returned for all gene-drug pairs where there is any evidence, irrespective of strength. Approximately 50 genes, related to 200 medicines.	
Return of results	How results are returned to healthcare professionals	Post	Categorical
		Email	
		Standalone Web-Portal	
		Directly into the Electronic Health Record (EHR)	
Chance of effectiveness	The average effectiveness of a treatment following pharmacogenetic testing	50% (baseline)	Continuous
		60%	
		70%	
		80%	
Chance of adverse drug reaction	The average risk of an ADR from treatment following pharmacogenetic testing	20% (baseline)	Continuous
		15%	
		10%	
		5%	
Turnaround time^a^	The time taken from requesting the test to receiving the results	5 days	Continuous
		10 days	
		15 days	
		20 days (baseline)	

*CLM* Conditional Logit Model, *GP* General Practitioner.

^a^Participants were made aware during the training material for the discrete choice experiment (DCE) that turnaround time represented the time from being seen in clinic by their GP to the respondent receiving their prescription. In these hypothetical scenarios, a prescription would not be issued prior to the pharmacogenetic results being available.

#### Experimental design

Based on the number of attributes and levels, an experimental design which directly compared every theoretical combination would result in 65,536 (*n* = (1^3^ × 4^4^)^2^) choice questions. These were rationalised to a manageable number using Ngene (ChoiceMetrics, Sydney, Australia). A subset (*n* = 16) of the possible choice sets (questions) was selected by generating a mathematical design minimising the predicted variance in the estimation of the coefficients. The surveys were created by splitting the 16 choice questions randomly into one of two blocks.

#### Survey design

The online survey included four sections: introduction; training material; choice questions; questions capturing demographic and attitudinal data (Supplementary Appendix 2). After the animated training material (Supplementary Appendix 3), respondents were randomised 1:1 to either the joint pain or low mood scenario. Within each scenario, respondents underwent a further 1:1 randomisation to either block 1 or block 2 of the choice questions. Each participant completed 8 choice questions in total.

### Data collection

#### Sample frame

The relevant study population was healthcare professionals who might be responsible for pharmacogenetic guided prescribing, were such a service available. General practitioners and pharmacists, working in primary care or community pharmacy settings, were identified as the relevant sample frame. The inclusion of pharmacists as a relevant sample reflects the changing scope of their clinical practice in the UK. Pharmacists can now train to be an independent prescriber with around one quarter of registered UK pharmacists currently licensed to prescribe [17]. In addition, from 2026, all newly qualified pharmacists will, on the day of their professional registration, be qualified independent prescribers [18]. Community pharmacies in England are also increasingly digitally enabled giving pharmacists access to view and update patients’ records within their GP system.

#### Sample size

There is no consensus on the best way to successfully estimate the sample size requirements for a DCE, though one commonly used approach is the rule of thumb of Orme (500*C [Max levels]/(Tasks*Alternatives)) which estimated that a sample size of at least 125 participants would be sufficient to estimate parameters for main effects analysis [19]. Larger sample sizes are required for sub-group analysis. The aim was to recruit a purposive sample of at least 100 participants per professional group.

#### Piloting

A quantitative pilot was undertaken with 30 individuals with experience of healthcare or healthcare related research, with 15 respondents completing each scenario. A conditional logit model was run on this dataset to assess whether the DCE had been designed and formatted appropriately. These responses were not included in the final analysis. Qualitative piloting was also undertaken (Supplementary Appendix 2).

#### Fielding

Participants were approached via email or online through professional organisations including the Royal College of General Practitioners (RCGP) and the Royal Pharmaceutical Society (RPS). Participants were asked to confirm their role within the survey and were disqualified if they did not meet eligibility. The survey was fielded from July until November 2023.

### Analysis

The presence of preference heterogeneity (representing how preferences varied) and scale heterogeneity (representing variation in randomness or error) was tested for using the methods suggested by Swait and Louviere (Supplementary Appendix 4) [20]. Once the datasets for analysis had been identified, a series of new models were estimated to identify the best functional form. A complete analysis plan is provided in Supplementary Appendix 5.

### Interpretation

Coefficients from the final selected model were used to estimate uptake in the context of different service designs. The “base-case” service represents how a pharmacogenetic testing service might be developed if testing was to be delivered using current infrastructure and approaches. The “base-case” model assumed results would be returned via post and are “focussed” in nature; reporting results for only the medicine which precipitated the request. It was estimated that turnaround time using current infrastructure and capacity would be 20 days.

Predicted uptake of the “base-case” service was then compared against a hypothetical “optimised” service. This represented a service template where each attribute had been augmented so that the level of each attribute with the largest positive coefficient from the final model was added simultaneously. For this “optimised” service, turnaround time was 10 days. The chance of effectiveness and chance of ADR were modelled across a spectrum, as the impact of pharmacogenetic testing on these attributes will vary depending on clinical context.

### Ethical approval

The study adhered to good clinical practice guidelines and the Declaration of Helsinki. It was approved by the University of Manchester Research Ethics Committee following proportionate review on 21 June 2023 (REF: 2023–16921). Informed consent was obtained from all subjects.

## Results

### The respondents

The analysis dataset included 235 completed surveys, 121 of which were completed by general practitioners (GPs) and 114 by pharmacists (Table 2). The majority, 90.2%, had no experience of pharmacogenetic guided prescribing. The median response time in the analysis dataset was 15.2 min.

**Table 2:** Participant characteristics.

Characteristic	General practitioners	Pharmacists	Complete dataset
	(*n* = 121)	(*n* = 114)	(*n* = 235)
Gender			
Male	49 (40.5%)	34 (29.8%)	83 (35.3%)
Female	71 (58.7%)	77 (67.5%)	148 (63.0%)
Non-binary	0 (0%)	2 (1.8%)	2 (0.9%)
Decline to answer	1 (0.8%)	1 (0.9%)	2 (0.9%)
Age (Years)			
Mean age	42.2	42.1	42.1
18–30	8 (6.6%)	13 (11.4%)	21 (8.9%)
30–40	52 (43.0%)	39 (34.2%)	91 (38.7%)
40–50	29 (24.0%)	31 (27.2%)	60 (25.5%)
50–60	20 (16.5%)	23 (20.2%)	43 (18.3%)
60–70	12 (9.9%)	7 (6.1%)	19 (8.1%)
70–80	0 (0.0%)	1 (0.9%)	1 (0.4%)
Ethnicity			
White British/White Irish	80 (66.1%)	84 (73.7%)	164 (69.8%)
Asian/Asian British	32 (26.5%)	21 (18.4%)	53 (22.6%)
Black/African/Caribbean	7 (5.8%)	4 (3.5%)	11 (4.7%)
Other ethnic group	1 (0.8%)	3 (2.6%)	4 (1.7%)
Mixed ethnicity	1 (0.8%)	2 (1.8%)	3 (1.3%)
Experience of pharmacogenetic guided prescribing			
Yes	4 (3.3%)	8 (7.0%)	12 (5.1%)
No	109 (90.1%)	103 (90.4%)	212 (90.2%)
Unsure	8 (6.6%)	3 (2.6%)	11 (4.7%)

Percentages rounded to the nearest 0.1% and therefore may not sum to 100%.

### Identification of preference heterogeneity

Preference and scale heterogeneity was identified between pharmacists and GPs, suggesting that preferences for a pharmacogenomic prescribing service are impacted by an individual’s role (Supplementary Appendix 4). The presence of preference or scale heterogeneity was not found between the two clinical scenarios (pain versus low mood). As such, the subsequent analysis was undertaken by separating data by role, but not by clinical scenario.

### Final econometric model

The best fit for both respondent datasets was found using an uncorrelated random parameter logit model (Table 3). This model assumed that the pre-specified continuous variables (Table 1) were linear and continuous (Supplementary Appendix 7 and 8).

**Table 3:** Random parameter logit model.

	General practitioners			Pharmacists		
Attribute	Estimated coefficient	95% confidence interval	*P* value	Estimated coefficient	95% confidence interval	*P* value
Type of data reported						
Focussed	−0.3	−0.53 to −0.07	<0.01	−0.35	−0.58 to −0.11	<0.01
Narrow	0.56	0.35 to 0.78	<0.01	0.24	0.01 to 0.47	0.04
Broad	−0.26	−0.50 to −0.02	0.01	0.11	0.44 to 0.22	0.12
Return of results						
Via post	−0.8	−1.09 to −0.50	<0.01	−0.78	−1.14 to −0.42	<0.01
Via email	−0.02	−0.28 to 0.24	0.89	−0.12	−0.38 to 0.15	0.38
Via web-portal	−0.1	−0.37 to 0.16	0.44	0.25	−0.07 to 0.56	0.13
Via EHR	0.92	0.61 to 1.23	<0.01	0.65	0.26 to 1.04	<0.01
Chance of effectiveness^a^	0.11	0.09 to 0.13	<0.01	0.12	0.10 to 0.15	<0.01
Chance of ADR^a^	−0.11	−0.14 to −0.08	<0.01	−0.11	−0.14 to −0.07	<0.01
Turnaround time^a^	−0.07	−0.10 to −0.04	<0.01	−0.1	−0.14 to −0.06	<0.01
Constant^b^	1.56	0.46 to 2.65	<0.01	3.03	1.74 to 4.32	<0.01

The Random Parameter Logit Model was estimated using categorical variables that were effects coded. Each estimated co-efficient represents the impact of that level on predicted uptake in the context of the mean effect of each attribute. *P* values assess whether including each level results in statistically significant (*p* < 0.05) difference from the average service, based on the mean effect of all other attributes.

^a^The estimated coefficients for the continuous variable represent the change in coefficient per unit (i.e. Percentage point increase (Chance of Effectiveness & Chance of ADR) or days (Turnaround Time)).

^b^The constant represents respondent’s preferences for some form of pharmacogenetic service vs receiving no pharmacogenetic test (i.e. opt out).

### Preferences for pharmacogenetic testing

Respondents consistently chose pharmacogenetic testing over opting for standard of care, as shown by the positive coefficient for the constant term in the estimated model (Table 3). Both professional groups preferred to have pharmacogenetic data reported as a “narrow” panel compared to other reporting strategies. “Focussed” reporting was associated with a negative co-efficient in both professional groups. Reporting data via a “broad” gene panel was associated with increased uptake for pharmacists, but not by GPs for whom it was associated with reduced uptake.

There was a preference in both professional groups for results to be returned within the EHR, particularly for GPs. Other than return via EHR, GPs had a negative preference for all other modalities of data return, including email and via a web-portal, whereas pharmacists were not averse to receiving results via a web portal. In both professional groups, return via either email or web-portal did not significantly impact uptake compared to the average service. These preferences were consistent across all effectiveness and ADR ranges (Supplementary Appendix 9). An increase in turnaround time led to reduced uptake. An increase in the ability of the pharmacogenetic test to improve medicines effectiveness, or reduce the rate of adverse drug reactions, were both associated with significantly increased uptake.

### Increase in predicted uptake from service optimisation

**Fig. 2:**
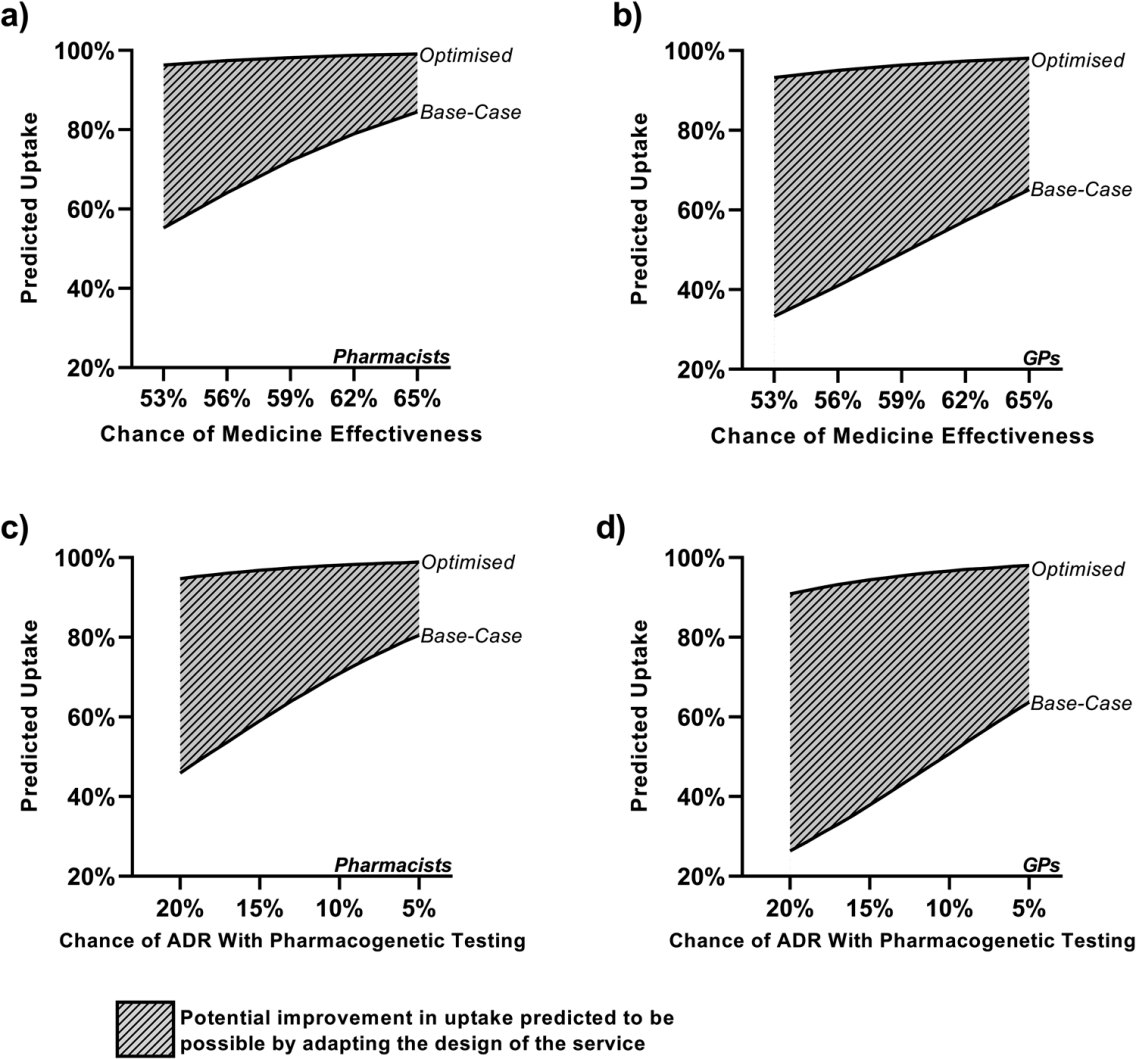
Differences in uptake between base-case & optimised service designs. A comparison of the predicted uptake of pharmacogenetic testing for the “base-case” and “optimised” services. Uptake is varied across an effectiveness and ADR range for both pharmacists and GPs. **a** Predicted uptake by pharmacists with changes in the chance of medicine effectiveness with pharmacogenetic testing. **b** Predicted uptake by GPs with changes in the chance of medicine effectiveness with pharmacogenetic testing. **c** Predicted uptake by pharmacists with changes in the chance of an adverse drug reaction with pharmacogenetic testing. **d** Predicted uptake by GPs with changes in the chance of an adverse drug reaction with pharmacogenetic testing.

The predicted uptake for the “base-case” service (Table 4) was relatively modest in both professional groups. Predicted uptake in this scenario was notably lower for GPs across the effectiveness and ADR continuum (Fig. 2). Adaptations to the “base-case” service meaningfully impacted predicted uptake for both professional groups, with GPs being most sensitive to these changes. At a chance of medicines effectiveness of 53%, representing an absolute improvement of 3% from prescribing without pharmacogenetics, the “base-case” service had an estimated uptake of 55.2% for pharmacists (Fig. 2a) and 33.3% for GPs (Fig. 2b). For the “optimised” service (Table 2), at the same improvement in effectiveness, the predicted uptake was and 96.3% and 93.3% for pharmacists and GPs, respectively. This represents a potential improvement of up to 41.1% in pharmacists and up to 59.9% in GPs from making changes to the design of the service.

**Table 4:** Base case and optimised services.

Attribute	“Base-case” service	“Optimised” service
Type of data reported	Focused	Narrow
Return of results	Post	Electronic health record
Chance of effectiveness	50%	Varied between 53 and 65%^a^
Chance of ADR	20%	Varied between 5 and 20%^a^
Turnaround time	20 days	10 days

The categorical levels chosen in the optimised service represent those with the highest co-efficient in the final selected model.

^a^Continuous variables were tested across a range of values. In the optimised service, when the chance of an ADR was varied, the chance of effectiveness was set at 50%. Conversely, when the chance of effectiveness was varied, the chance of an ADR was set at 20%.

## Discussion

This study aimed to quantify the preferences of a sample of GPs and pharmacists for the design of a pharmacogenetic testing service intended for primary care in the NHS. The findings show respondents preferred pharmacogenetic testing over no testing. However, both professional groups, and especially GPs, were highly sensitive to service design, with predicted uptake varying considerably depending on the service offered. No difference in preferences was identified based on the clinical scenario, though a difference was found based on professional role. This represents the first study to quantify the views of two professional groups, able to prescribe medicines, to inform the development of a pharmacogenetic service in primary care.

In both professional groups, respondents preferred to receive pharmacogenetic results via a “narrow” gene panel, rather than a “focussed” single result or a “broad” panel. With an average clinic appointment time of 10 min, it is understandable that GPs preferred results that were immediately actionable [21]. Any requirement to consider the level of evidence and actionability for individual genes and variants, as might be required with a “broad” panel, is unlikely to integrate into an already complex and time-sensitive environment [22].

Though pharmacists also preferred “narrow” results, they were not significantly opposed to receiving more detailed data. This may reflect their distinct role in leading the medicines optimisation process where there may be relatively greater scope to consider these less definitive findings. It may also reflect differences in training, with pharmacists potentially having more exposure to pharmacogenetics education during their undergraduate curricula [23]. Indeed, pharmacist-led medication reconciliation programmes, augmented by pharmacogenetics, have been successfully implemented into routine practice in other health systems [4, 24, 25]. This suggests there is space for a dual model of pharmacogenetic implementation in primary care, with pharmacists and GPs playing distinct but complementary roles.

Both professional groups strongly preferred services where results were returned directly within the EHR and, conversely, were more likely to reject services where results were returned via post or email. There are a range of different approaches to how pharmacogenetic data might be stored and surfaced within an EHR from basic solutions such as returning a portable document format file (PDF), to more advanced solutions such as creating clinical decision support (CDS) notifications [26, 27]. Prescribing support software tools are already commonly used in UK general practice to surface CDS alerts and prompts during the prescribing workflow based on patients test results, but these are not in place for pharmacogenetic data [28]. Any solution which necessitates messaging data across institutional boundaries, from laboratory to GP practice, will require the development of technical systems and data standards to facilitate interoperability and clinical interpretation [29]. Compared to other areas of the NHS, General Practice is significantly more advanced with respect to interoperable IT infrastructure, with long established links between practices and pathology departments [30, 31]. However, no comparable system exists for genomic data. These findings suggest that this should be addressed as a priority if pharmacogenetics is to be implemented in routine clinical practice.

These results show that the design of a pharmacogenetic service materially impact predicted uptake and is as relevant as the potential prescribing improvements offered by the test itself. The ability of a pharmacogenetic test to improve safety or improve effectiveness will vary depending on the clinical context, including the baseline risk without testing. The PREPARE study, an implementation trial of a “narrow” 12-gene pharmacogenetic panel across seven European countries, found a reduction of clinically relevant ADRs of up to 30% [6]. The findings from this DCE suggest that a reduction in ADRs alone is not sufficient to foster widespread uptake amongst both professional groups, but especially GPs. Delivering any pharmacogenetic intervention using current (“baseline”) infrastructure would result in imperfect uptake, something which should be reflected in any health-economic modelling, but has been neglected in many previous analyses [32, 33].

DCEs represent an approach to measure choices and infer stated preferences and, although estimates suggest they produce reliable predictions, they are susceptible to hypothetical bias [34]. Given current pressures experienced within primary care, even if an optimised service were developed, it is possible that pharmacogenetic testing might not be routinely requested when considered amongst the hierarchy of demands experienced by GPs and pharmacists on a daily basis. It should also be noted that any survey can be subject to sampling bias, and the stakeholders who completed the survey may not be fully representative of the primary care population as a whole with only those already interested in pharmacogenetics completing the survey. Additionally, these findings may not be generalisable to other healthcare professionals or healthcare settings, and independent replication would be required.

These findings highlight which aspects of a service might impact uptake, which can be used to guide design. However, stated preferences are unlikely to identically match revealed preferences, especially as other factors not tested here such as access to testing, reimbursement mechanisms, and availability of guidance are also likely to all influence uptake. As such, any changes made to services based on these findings should be tested in practice, using revealed preference methodologies, prior to wide-spread implementation.

There is a high level of acceptability for pharmacogenetic testing in primary care, but this is contingent on the service being designed appropriately. As such, implementation programmes should focus not only on making testing available but consider the full clinical pathway including digital and data interoperability. This will require dedicated resource and expertise, requiring the involvement of clinical stakeholders from the outset. The organisational challenges and financial investment to achieve this at scale will be substantial, but these findings suggest that the potential rewards are considerable.

## Supplementary information

Supplemental Material

Survey (Depression)

Survey (Joint Pain)

## Data Availability

All survey materials and the final models are made available within the supplementary appendix.
