# Supplementary material for: Understanding general practitioner and pharmacist preferences for pharmacogenetic testing in primary care: a discrete choice experiment
Source: Pharmacogenomics J. 2024 Aug 9;24(5):25. doi: 10.1038/s41397-024-00344-z (PMC11315669; doi:10.1038/s41397-024-00344-z)
Supplement: Supplementary file 1 — Supplemental Material [file 41397_2024_344_MOESM1_ESM.docx]

**Supplementary Information: Understanding General Practitioner and Pharmacist Preferences for Pharmacogenetic Testing in Primary Care: A Discrete Choice Experiment**

**Contents**

1. **Appendix 1: Establishing Attributes and Levels**
2. **Appendix 2: Final Version of the Survey**
3. **Appendix 3: Survey Animation**
4. **Appendix 4: Swait and Louviere Plot to Assess for Heterogeneity**
5. **Appendix 5: Analysis Plan**
6. **Appendix 6: Baseline Conditional Logit Model for Each Professional Group and Whole Dataset**
7. **Appendix 7: Baseline Conditional Logit Model Effects Coded for Continuous Attributes with Visual Inspection of Coefficient Slope**
8. **Appendix 8: Comparison of different models to Identify Best Fit**
9. **Appendix 9A: Predicted Uptake by Type of Data Reported.**
10. **Appendix 9B: Predicted Uptake by Method for Returning Results**

**Appendix 1 – Establishing Attributes and Levels**

We previously undertook a systematic scoping review of the pharmacogenetic literature to identify the key attributes of a pharmacogenetic service. This work has been published but, in brief, a bidirectional citation search was used to identify literature of interest.^1^ A starting pool of “initial pearls” was identified via a pragmatic Boolean search of Medical Subject Heading (MeSH) Terms within Web of Science; MEDLINE (via OVID); Embase (via OVID); and PubMed Central on 1 February 2022. The search [(((Pharmacogenetics) OR (Pharmacogenomics)) AND (Implementation)) AND (pre-emptive)] was used. Four screening rounds were required to complete the bidirectional citation search which involved the review of 8,355 abstracts and 104 relevant publications were identified for analysis.

A mixed methods qualitative sub-study was conducted to inform the selection and wording of attributes and levels. We utilised ethnographic observations, user research workshops and prototyping methods in this study. The participants for this study included general practitioners and pharmacists. In total we undertook five sessions of ethnographic observation to understand current practices and workflows. This was followed by three user research workshops, each with their own topic guide starting with personas and early ideation, through to exploring the potential attitudes towards a pharmacogenomic service. We subsequently analysed workshop data using affinity diagramming and refined the key requirements for a pharmacogenomic service collaboratively as a multi-disciplinary project team. This allowed us to draw out key attributes and associated levels, to use within the discrete choice experiment (DCE).

The way in which the data was returned, and the amount of data returned, were consistently reported as important design considerations. The time it took to receive results was considered important and represented a continuous “value” attribute within the DCE, allowing calculation of marginal rates of substitution (MRS) in terms of respondents’ willingness to wait (WTW) for a test. The ability of the test itself to reduce rates of adverse drug reactions (ADRs) and improve medicines effectiveness, were also considered important. Both out of pocket and NHS service costs were not included as attributes. It was decided that cost would not be an attribute as there are several costing models which could be operated in the NHS, and individual GPs would not be expected to consider costs per patient in a prescribing moment. The levels for each attribute were set to represent clinically meaningful options and reflected potentially viable options which could be offered within a new pharmacogenetic testing service. This was based on the experience of the clinical members of the research team (JM, WGN, VS, JK).

**Appendix 2 – Final Survey**

The survey comprised four sections:

1. The study background introducing pharmacogenetics.
2. Training material.
3. The choice questions (See Example below).
4. Background information on respondents and their attitudes towards the use of genetic data.

A final version of both survey versions has been provided as separate supplementary files which are accessible online (See supplementary survey files).

An animation (Appendix 3) was used to deliver an element of the introductory and training material. Previous studies have demonstrated that using animated training materials do not change the preferences of respondents, but may improve choice consistency.^3^ The survey was presented in English and programmed using HTML for online administration using SSI Web 8.3.8 Sawtooth software.

The final survey underwent a quantitative pilot, described in the main manuscript. However, beforehand, a qualitative pilot was also undertaken with healthcare professionals (n=5) with no prior experience of pharmacogenetic guided prescribing. Participants were asked to complete the survey and describe what they were doing using “think aloud” methodology. A researcher (JHM) observed the surveys being completed and made note of A) technical issues, B) uncertainty on the instructions and c) difficulties distinguishing between the service designs. Participants were generally able to complete the survey without issue, but small adaptations were made to the figures (see example below) within the choice sets based on this feedback.

**Supplementary Survey File Legends**

**Survey (Depression):** Example of the survey participants were asked to complete if randomized to the depression arm.

**Survey (Joint Pain):** Example of the survey participants were asked to complete if randomized to the joint pain arm

**Example of a question in the discrete choice experiments.**

**
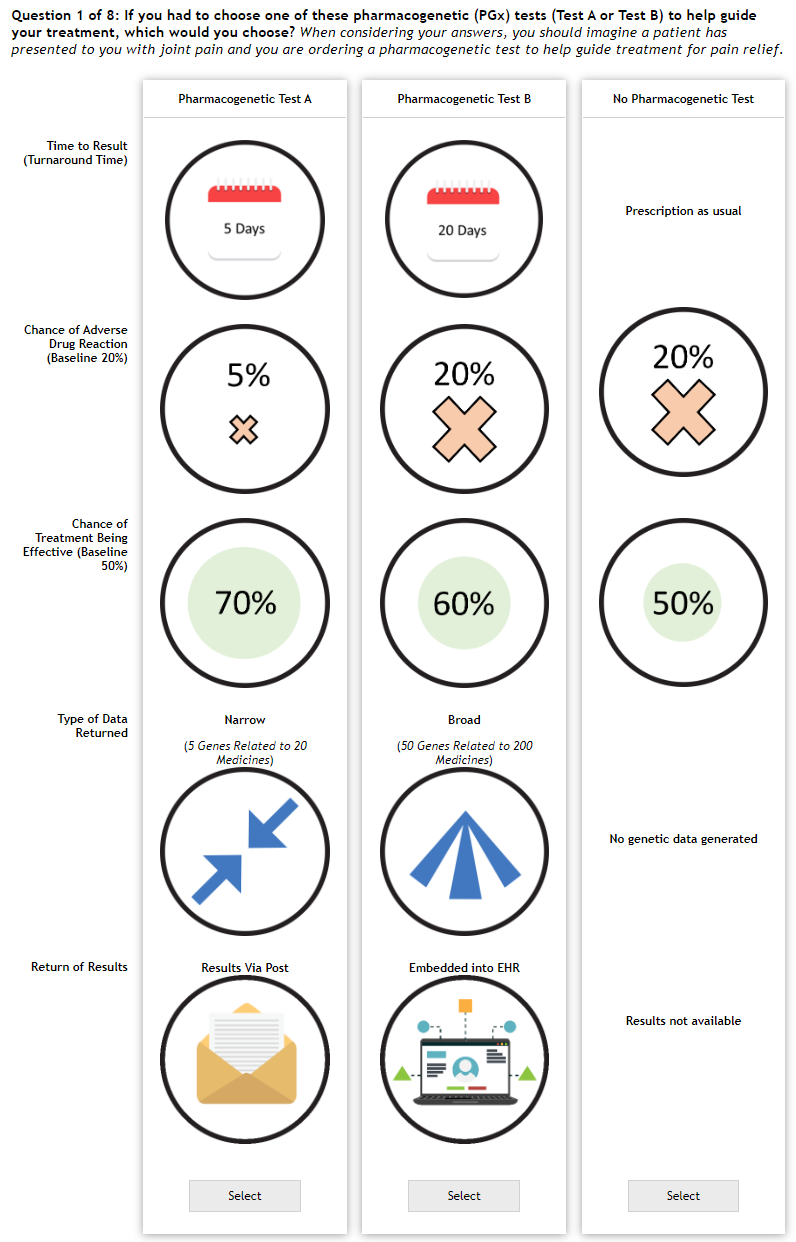
**

**Appendix 3 – Final Animation**

The final animation has been published online (https://doi.org/10.48420/25218194.v1)

**Appendix 4: Analysis to Assess for the Presence of Scale Heterogeneity**

As there were two versions of the survey (pain; low mood) and two sets of respondents (GPs and Pharmacists), it was necessary to determine if scale heterogeneity was present. If scale heterogeneity was not present, then the results of the two survey versions and/or two sets of respondents could be combined into one dataset for analysis.

The coefficients for each scenario were plotted following the recommendations of Swait and Louivere (Figure S1 and S2).^4^ A line of best fit was then estimated for the plot of coefficients, and the R^2^ and slope of the line of best fit was assessed for evidence of scale heterogeneity between the scenarios. This analysis did not identify the presence of scale heterogeneity, with a R^2^ of 0.824 and an intercept of y=0.922x – 0.0167. This suggested that preference was not impacted by the exemplar clinical scenario (i.e., pain vs low mood).

**Figure S1: Plot to assess for the presence of scale heterogeneity between versions.**


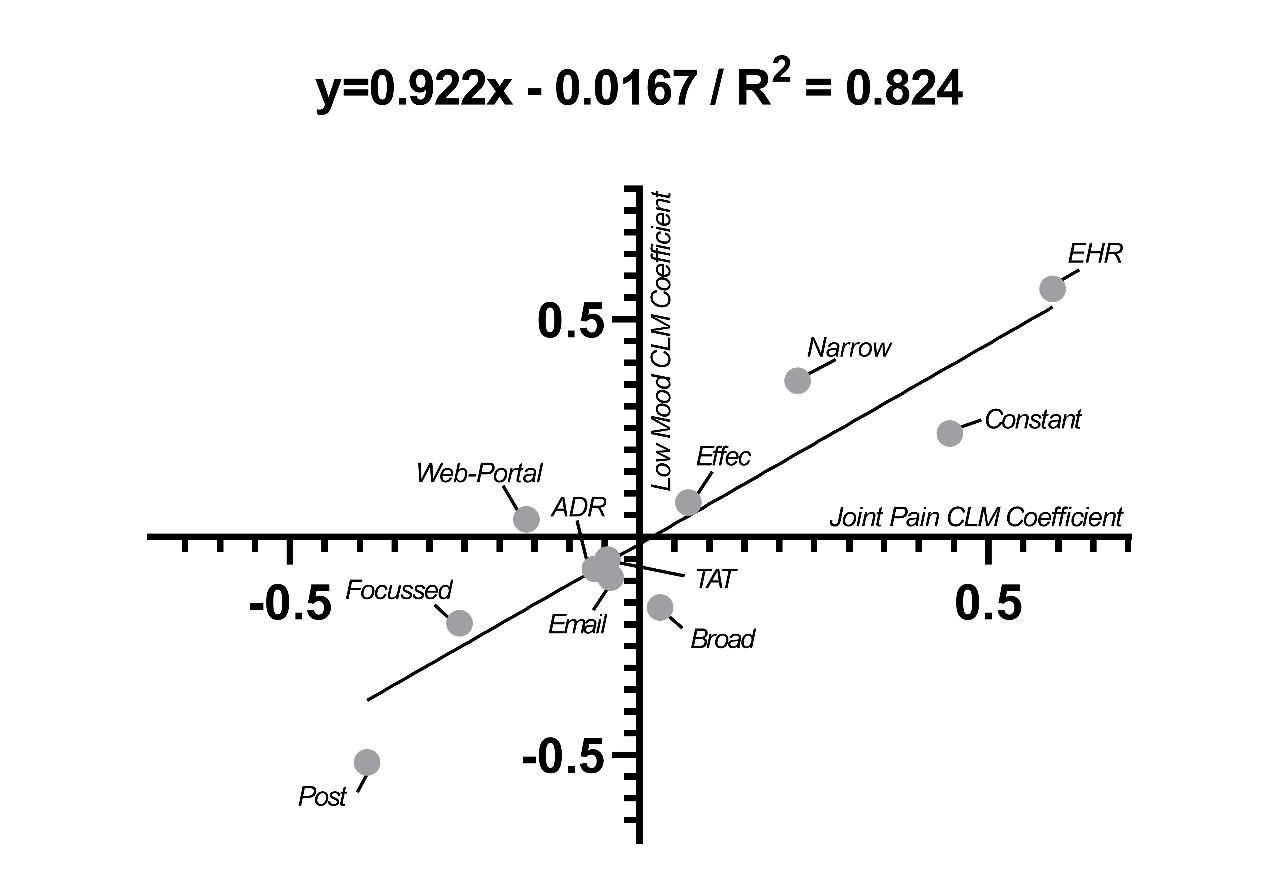


**Footnote:** CLM = Conditional logit Model. TAT= Turnaround Time. EHR = Electronic Health Record. ADR = Chance of Adverse Drug Reaction. Effec=Chance of Effectiveness.

Comparison coefficients by professional group (Figure S5.2) did identify scale heterogeneity, with an R^2^ of 0.0835 and an intercept of y=0.187x – 0.0455. This suggested that preference was influenced by professional role.

**Figure S2: Plot to assess for the presence of scale heterogeneity between professional groups.**


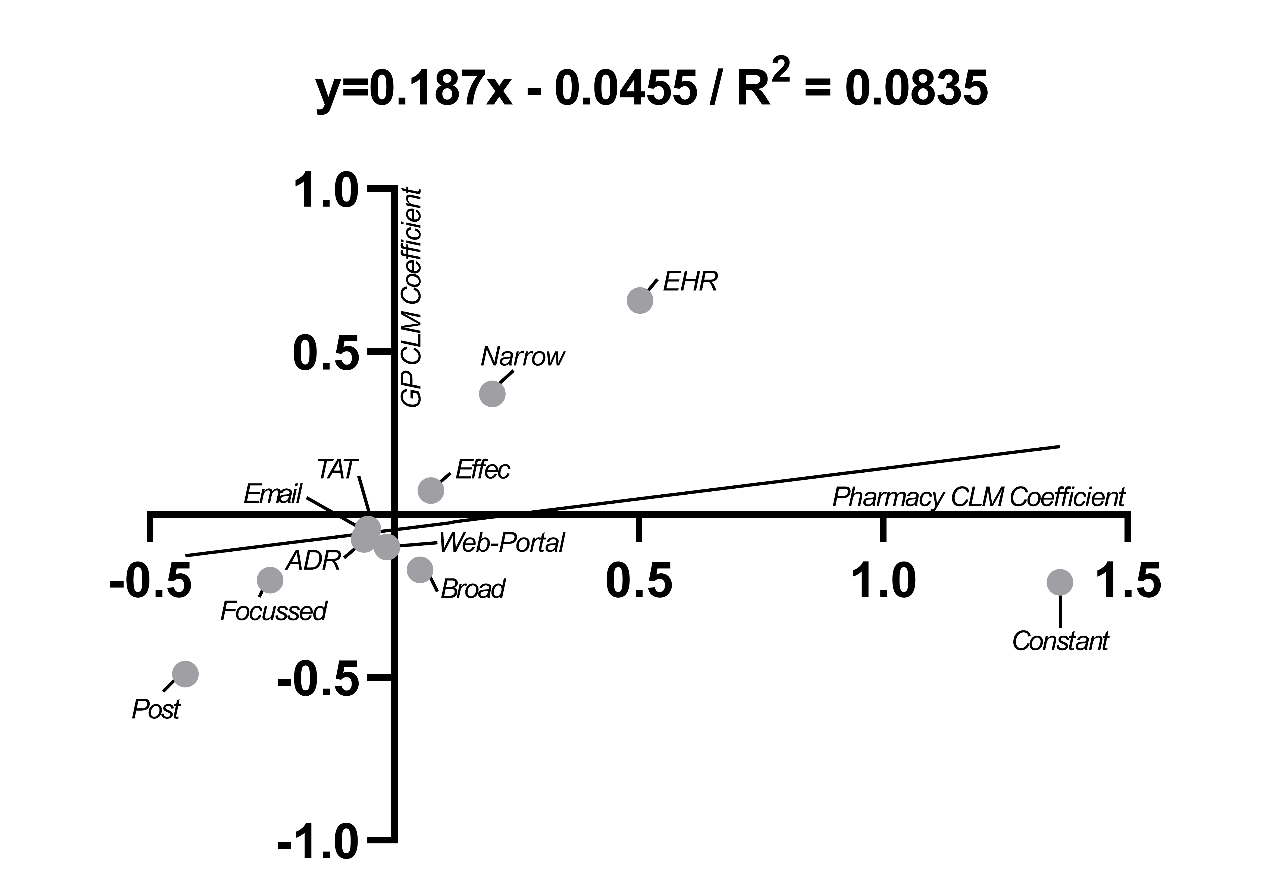


**Footnote:** CLM = Conditional logit Model. TAT= Turnaround Time. EHR = Electronic Health Record. ADR = Chance of Adverse Drug Reaction. Effec=Chance of Effectiveness. GP = General Practice

**Appendix 5 – Analysis Plan**

**Analysis Approach**

Individual choice responses were used as the dependent variable in the model. A conditional logit model was created for the whole dataset in the first instance and the sign of the coefficient for each attribute indicated the direction of preference for each attribute and level. Qualitative variables were effects coded and the continuous variables were treated as linear in the baseline analysis. Four distinct conditional logit models were first developed by splitting the data by each of the two scenarios (pain and low mood) and by the role of the respondent (GP and Pharmacists).

A pre-specified minimum completion time of 5 minutes was established, based on observing colleagues with no prior awareness of pharmacogenetics or DCEs, complete the survey. Any survey completion time under 5 minutes was considered too rapid to have meaningfully digested the introductory material and completed the survey and were excluded from the analysis.

**Identification of preference Heterogeneity**

The presence of preference heterogeneity (representing how preferences varied) and scale heterogeneity (representing variation in randomness or error) was tested for using the methods suggested by Swait and Louviere (Appendix 4).^21^ If preference and scale heterogeneity existed, then the datasets were not merged, and analysis was undertaken separately. If there was no evidence of preference and scale heterogeneity, preferences for the test would be deemed to be the same across clinical scenarios and respondents, and the data combined. Once the datasets for analysis had been identified, a series of new models were estimated to identify the best functional form by comparing Akaike’s information criterion (AIC) and the Bayesian Information Criterion (BIC) across models (Appendix 5: Analysis Plan).

**Model Selection**

Model selection was determined by comparing Akaike’s information criterion (AIC) and the Bayesian Information Criterion (BIC) across models. Initially, models were compared using either a single constant to represent a preference for receiving any tests versus opting out or separate constants for Test A or Test B versus no testing. The inclusion of two constants tests for a tendency for respondents to disproportionately choose the left- or right-hand option regardless of the attributes and levels. Several models including non-linear specifications of the turnaround time attribute were tested using AIC and BIC to choose the best fitting model. These models included quadratic, logarithmic and piecewise approaches. Upon selection of the final functional form, uncorrelated and fully correlated random parameter logit models were used to allow for preference heterogeneity and preference and scale heterogeneity respectively.

**Appendix 6: Baseline Conditional Logit Models**

1. Baseline conditional logit model for whole dataset (fixed effects)

| choice | Coef. | Std. Err. | z | P>z | [95% Conf. | Interval] |
| --- | --- | --- | --- | --- | --- | --- |
| focused | -0.22766 | 0.051953 | -4.38 | 0 | -0.32949 | -0.12583 |
| narrow | 0.290158 | 0.054356 | 5.34 | 0 | 0.183622 | 0.396693 |
| Broad | -0.0625 | 0.0553781 | -1.13 | 0.259 | -0.17103 | 0.046041 |
| viapost | -0.45499 | 0.068652 | -6.63 | 0 | -0.58954 | -0.32043 |
| viaemail | -0.06456 | 0.062481 | -1.03 | 0.301 | -0.18702 | 0.057899 |
| webportal | -0.06319 | 0.069909 | -0.9 | 0.366 | -0.20021 | 0.073826 |
| EHR | 0.582743 | 0.0746823 | 7.8 | 0 | 0.436638 | 0.729117 |
| chanceeff | 0.074199 | 0.003555 | 20.87 | 0 | 0.067231 | 0.081167 |
| chanceadr | -0.06813 | 0.005681 | -11.99 | 0 | -0.07927 | -0.057 |
| tat | -0.04878 | 0.006971 | -7 | 0 | -0.06244 | -0.03512 |
| asc | 0.387474 | 0.130859 | 2.96 | 0.003 | 0.130995 | 0.643953 |

1. Baseline conditional logit model for pharmacy dataset (fixed effects)

| choice | Coef. | Std. Err. | z | P>z | [95% Conf. | Interval] |
| --- | --- | --- | --- | --- | --- | --- |
| focused | -0.2539 | 0.074811 | -3.39 | 0.001 | -0.40052 | -0.10727 |
| narrow | 0.200784 | 0.078382 | 2.56 | 0.01 | 0.047158 | 0.35441 |
| Broad | 0.053114 | 0.080608 | 0.66 | 0.510 | -0.104875 | 0.211102 |
| viapost | -0.42705 | 0.099181 | -4.31 | 0 | -0.62144 | -0.23266 |
| viaemail | -0.06087 | 0.089238 | -0.68 | 0.495 | -0.23578 | 0.114029 |
| webportal | -0.01472 | 0.101692 | -0.14 | 0.885 | -0.21403 | 0.184595 |
| Elec Health | 0.502647 | 0.1104532 | 4.5 | 0 | 0.286162 | 0.719131 |
| chanceeff | 0.07468 | 0.005119 | 14.59 | 0 | 0.064646 | 0.084714 |
| chanceadr | -0.06207 | 0.008055 | -7.71 | 0 | -0.07786 | -0.04628 |
| tat | -0.05386 | 0.010242 | -5.26 | 0 | -0.07393 | -0.03379 |
| asc | 1.361366 | 0.222533 | 6.12 | 0 | 0.92521 | 1.797521 |

1. Baseline conditional logit model for GP dataset (fixed effects)

| choice | Coef. | Std. Err. | z | P>z | [95% Conf. | Interval] |
| --- | --- | --- | --- | --- | --- | --- |
| focused | -0.20133 | 0.072583 | -2.77 | 0.006 | -0.34359 | -0.05907 |
| narrow | 0.371249 | 0.07622 | 4.87 | 0 | 0.221861 | 0.520638 |
| Broad | -0.16992 | .0776579 | -2.19 | 0.029 | -0.322128 | -0.017714 |
| viapost | -0.48981 | 0.096543 | -5.07 | 0 | -0.67903 | -0.30059 |
| viaemail | -0.06867 | 0.088365 | -0.78 | 0.437 | -0.24186 | 0.104522 |
| webportal | -0.0994 | 0.097073 | -1.02 | 0.306 | -0.28966 | 0.090858 |
| Elec Health | 0.657882 | 0.102078 | 6.44 | 0 | 0.457812 | 0.857950 |
| chanceeff | 0.074373 | 0.005005 | 14.86 | 0 | 0.064564 | 0.084182 |
| chanceadr | -0.07523 | 0.00814 | -9.24 | 0 | -0.09118 | -0.05927 |
| tat | -0.04458 | 0.009639 | -4.63 | 0 | -0.06348 | -0.02569 |
| asc | -0.20811 | 0.17765 | -1.17 | 0.241 | -0.55629 | 0.140079 |

**Appendix 7: Baseline Conditional Logit Model Effects Coded for Continuous Attributes with Visual Inspection of Coefficient Slope**

1. Baseline Conditional Logit Model Effects Coded for Turnaround Time with (B) Visual Inspection of Turnaround Time Slope.

| choice | Coef. | Std. Err. | z |
| --- | --- | --- | --- |
| focused | -0.22087 | 0.05766 | -3.83 |
| narrow | 0.278999 | 0.065384 | 4.27 |
| viapost | -0.45784 | 0.077982 | -5.87 |
| viaemail | -0.06735 | 0.062678 | -1.07 |
| webportal | -0.05565 | 0.070851 | -0.79 |
| chanceeff | 0.073709 | 0.003634 | 20.29 |
| tat | -0.04976 | 0.008795 | -5.66 |
| **5 Days** | -0.05537 | 0.160667 | -0.34 |
| **10 Days** | 0.075067 | 0.148154 | 0.51 |
| **15 Days** | -0.34695 | 0.167143 | -2.08 |
| **20 Days** | -0.74221 | 0.186024 | -3.99 |

**Figure S3: Change in co-efficient by effects coded turnaround time**

**
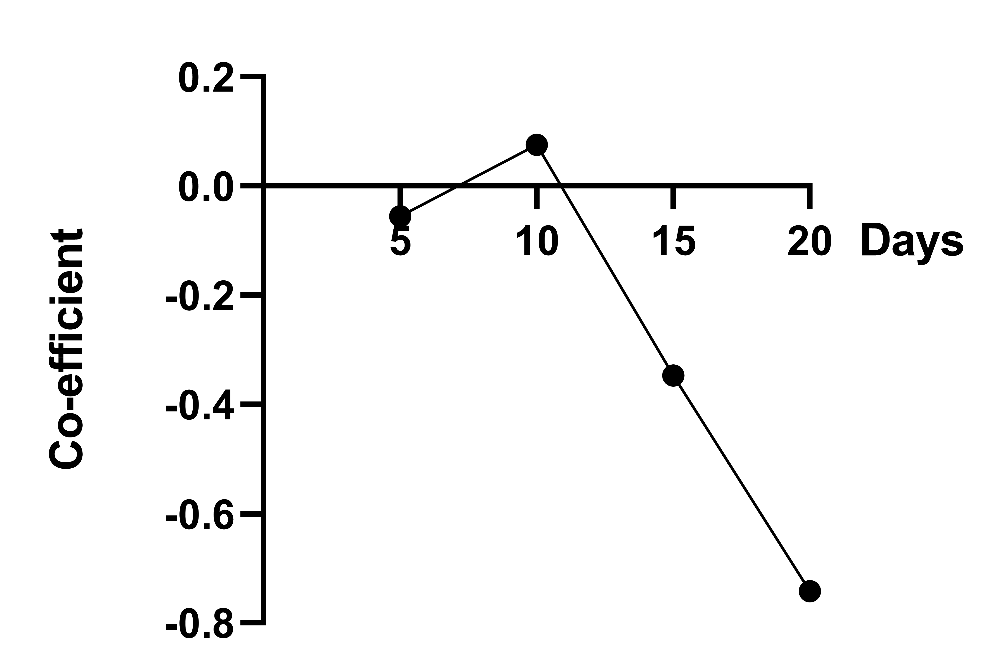
**

1. Baseline Conditional Logit Model Effects Coded for Chance of Effectiveness with (D) Visual Inspection of Chance of Effectiveness Slope

| choice | Coef. | Std. Err. | z |
| --- | --- | --- | --- |
| focused | -0.22087 | 0.05766 | -3.83 |
| narrow | 0.278999 | 0.065384 | 4.27 |
| viapost | -0.45784 | 0.077982 | -5.87 |
| viaemail | -0.06735 | 0.062678 | -1.07 |
| webportal | -0.05565 | 0.070851 | -0.79 |
| chanceeff | 0.073709 | 0.003634 | 20.29 |
| tat | -0.04976 | 0.008795 | -5.66 |
| **5%** | **0.508506** | **0.109244** | **4.65** |
| **10%** | **0.247372** | **0.112995** | **2.19** |
| **15%** | **-0.05237** | **0.112479** | **-0.47** |
| **20%** | **0.178497** | **0.083605** | **2.14** |

**Figure S4: Change in co-efficient by effects coded Chance of Effectiveness**

**
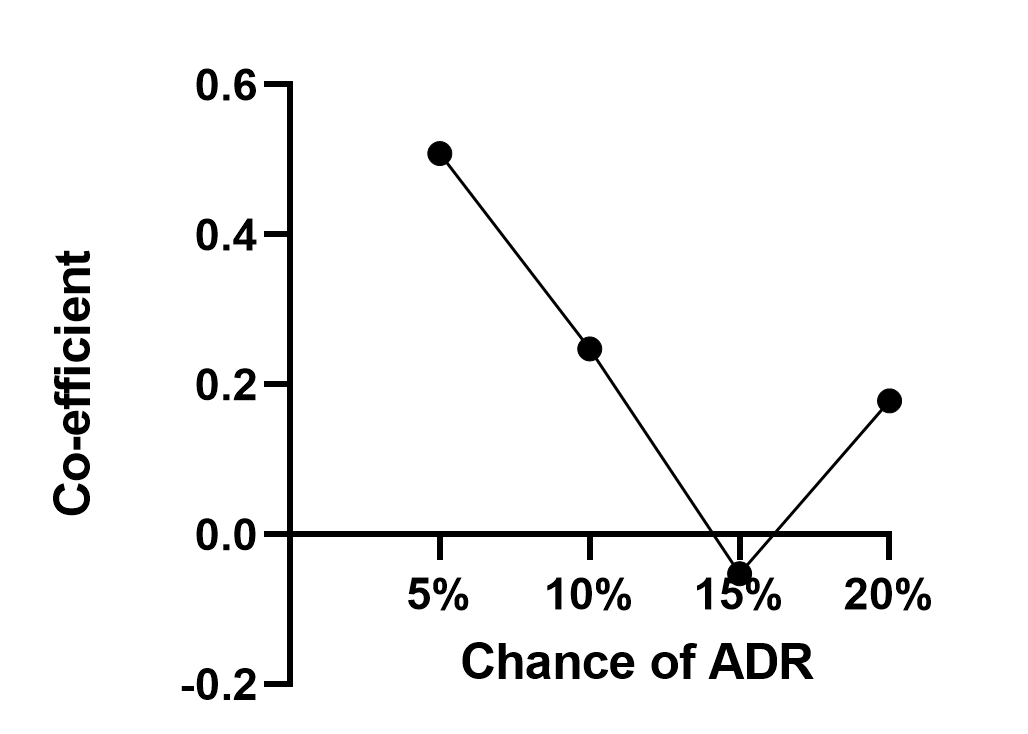
**

(E) Baseline Conditional Logit Model Effects Coded for Chance of ADR with (F) Visual Inspection of Chance of Adverse Drug Reaction Slope.

| choice | Coef. | Std. Err. | z |
| --- | --- | --- | --- |
| focused | -0.2227 | 0.052689 | -4.23 |
| narrow | 0.283883 | 0.055326 | 5.13 |
| viapost | -0.45306 | 0.068864 | -6.58 |
| viaemail | -0.04281 | 0.073089 | -0.59 |
| webportal | -0.05003 | 0.071866 | -0.7 |
| chanceadr | -0.0626 | 0.010202 | -6.14 |
| tat | -0.04959 | 0.007099 | -6.99 |
| **50%** | **0.243202** | **0.099958** | **2.43** |
| **60%** | **-0.38619** | **0.109636** | **-3.52** |
| **70%** | **0.467153** | **0.12326** | **3.79** |
| **80%** | **1.16404** | **0.107911** | **10.79** |

**Figure S5: Change in co-efficient by effects coded Chance of Effectiveness**

**
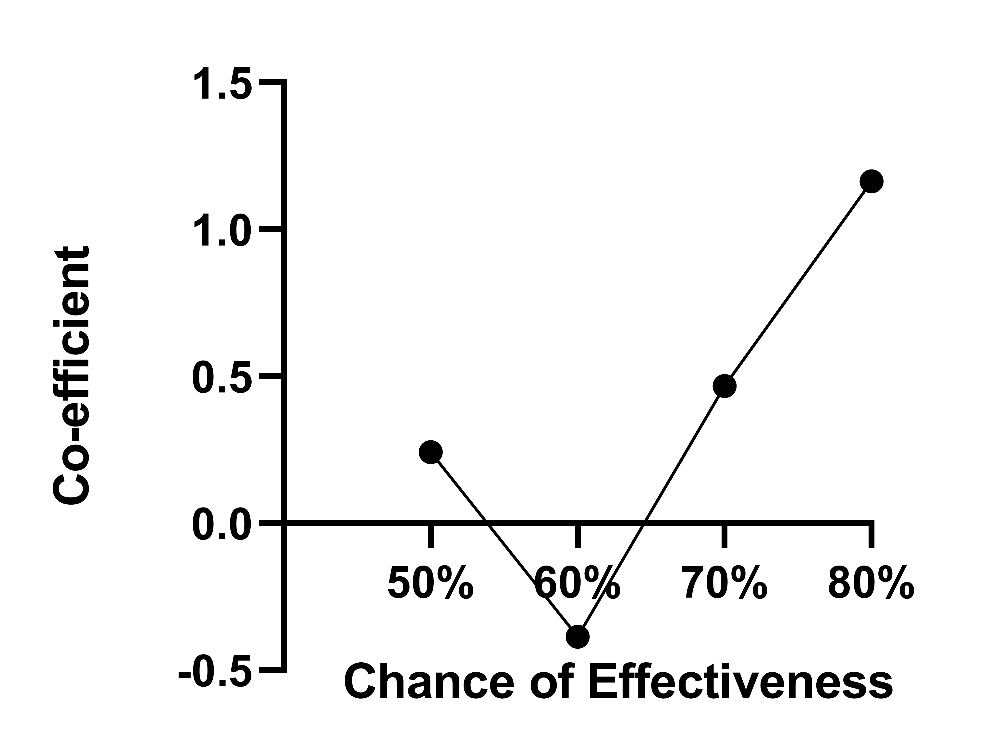
**

**Appendix 8: Comparison of different models to Identify Best Fit**

| **Whole Dataset** | | | |
| --- | --- | --- | --- |
|  | LL | AIC | BIC |
| **CLM [Linear + 1 Constant]** | -1263.89 | 2545.779 | 2605.479 |
| **CLM [Linear + 2 Constants]** | -1258.93 | 2537.85 | 2604.184 |
| **CLM [Quadratic (TAT)]** | -1262.62 | 2545.237 | 2611.571 |
| **CLM [Effects coded (TAT)]** | -1261.45 | 2544.893 | 2617.86 |
| **CLM [Effects coded (Effectiveness)]** | -1263.65 | 2549.304 | 2622.271 |
| **CLM [Effects coded (ADR)]** | -1263.65 | 2549.308 | 2622.275 |
| **CLM [Log Model]** | -1265.44 | 2548.874 | 2608.575 |
| **CLM [Piecewise TAT 10]** | -1261.45 | 2542.903 | 2609.236 |
| **CLM [Piecewise TAT 15]** | -1263.89 | 2547.778 | 2614.112 |
| **Random Parameter Logit Model (Uncorrelated)**  [Selected Final Model] | -1052.17 | 2140.343 | 2259.744 |

**Appendix 9A: Predicted Uptake by Type of Data Reported.
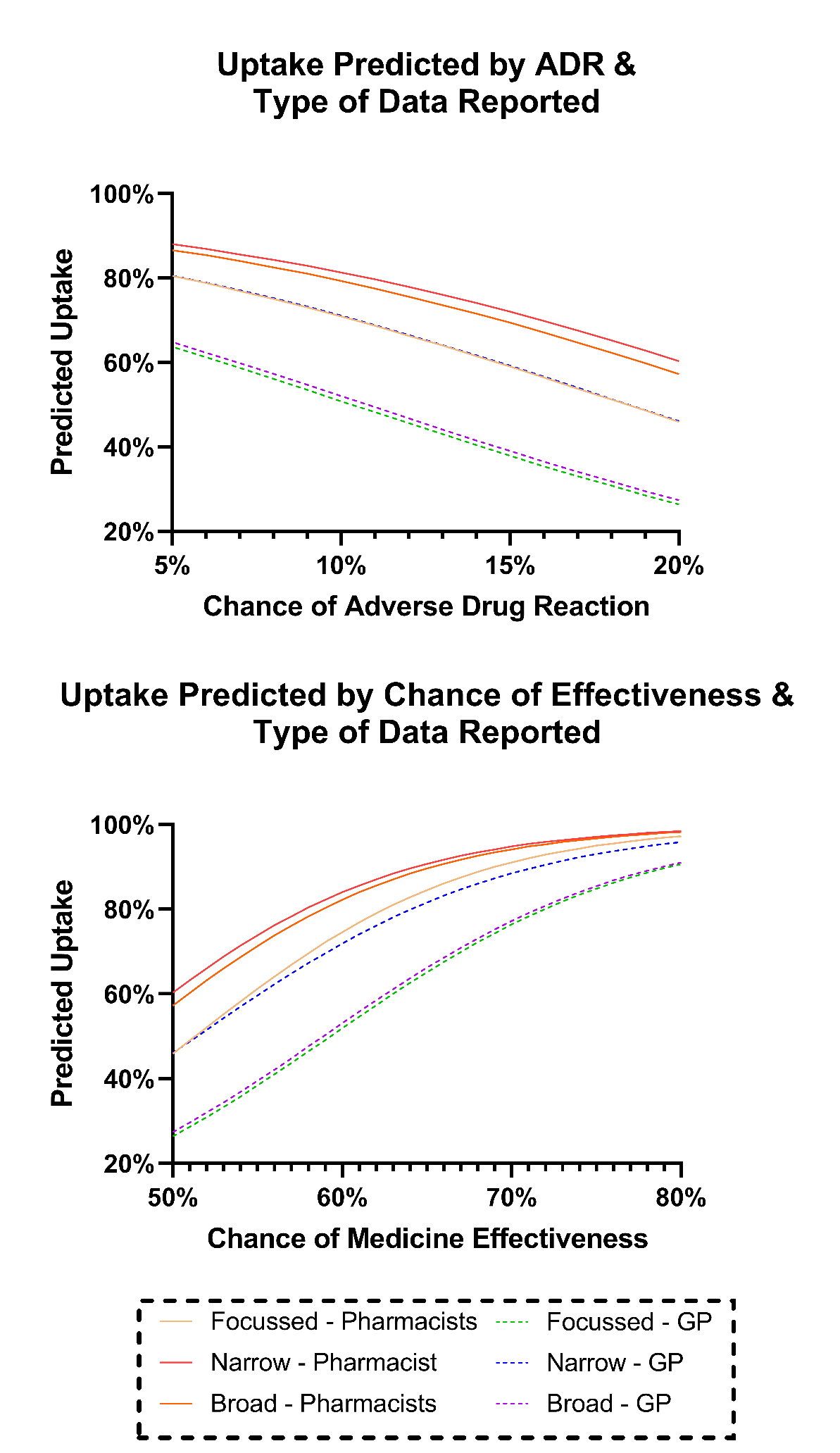
**

**Appendix 9B: Predicted Uptake by Method for Returning Results
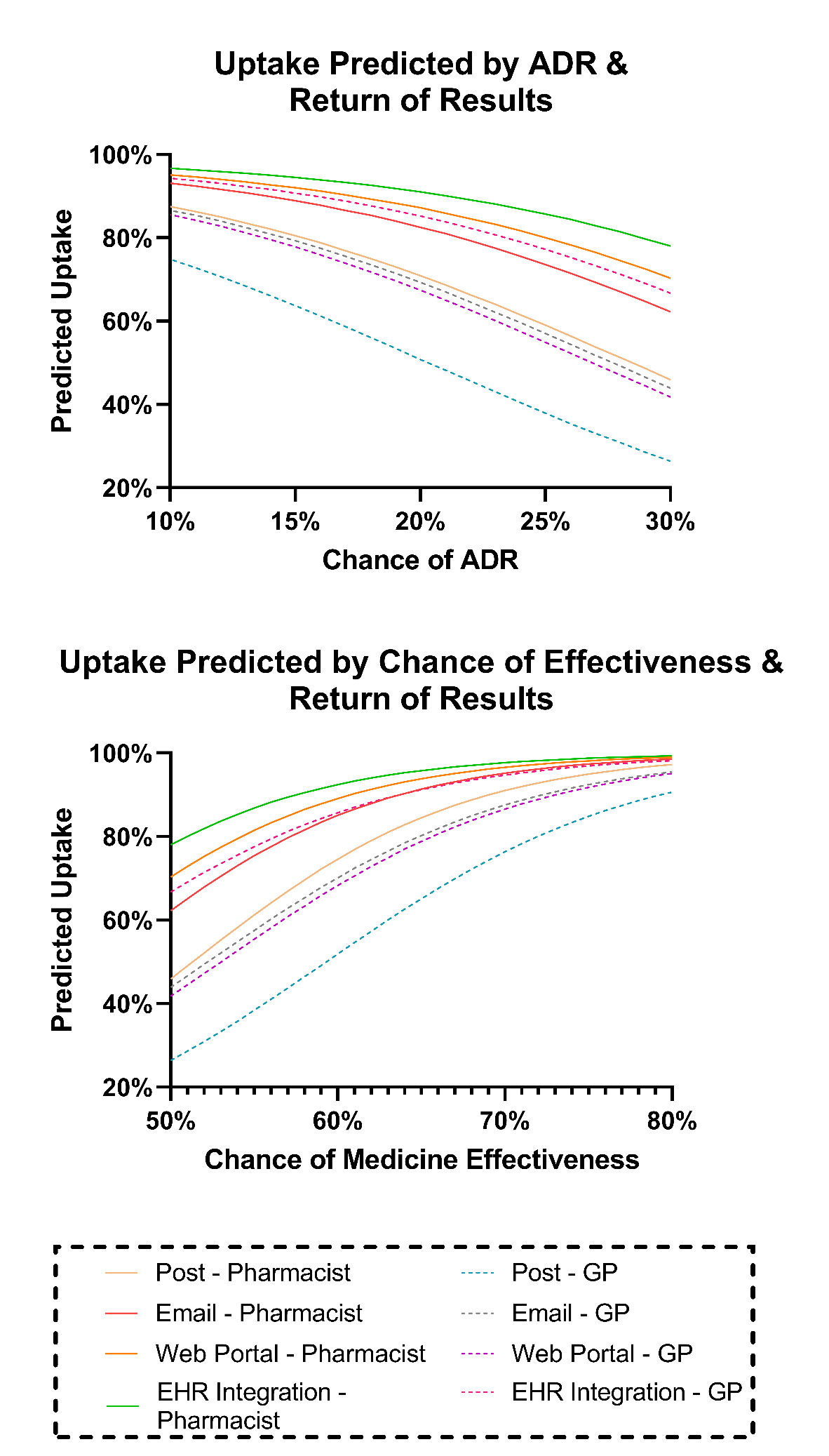
**

**Supplementary References**

1. McDermott JH, Wright S, Sharma V, Newman WG, Payne K, Wilson P. Characterizing pharmacogenetic programs using the consolidated framework for implementation research: A structured scoping review. Front Med 2022;9:945352.

2. Vass C, Rigby D, Payne K. The Role of Qualitative Research Methods in Discrete Choice Experiments. Med Decis Making 2017;37(3):298–313.

3. Vass CM, Davison NJ, Vander Stichele G, Payne K. A Picture is Worth a Thousand Words: The Role of Survey Training Materials in Stated-Preference Studies. The Patient 2020;13(2):163–73.

4. Swait J, Louviere J. The Role of the Scale Parameter in the Estimation and Comparison of Multinomial Logit Models. J Mark Res 1993;30(3):305–14.
