# Supplementary material for: Understanding general practitioner and pharmacist preferences for pharmacogenetic testing in primary care: a discrete choice experiment
Source: Pharmacogenomics J. 2024 Aug 9;24(5):25. doi: 10.1038/s41397-024-00344-z (PMC11315669; doi:10.1038/s41397-024-00344-z)
Supplement: Supplementary file 2 — Survey (Depression) [file 41397_2024_344_MOESM2_ESM.pdf]

Login

Thankyou for you interest in this survey. Please click "Next" to begin.

Note:

Your survey contains link variables. They are displayed below for testing purposes only. This message and the fields below will not appear in the regular survey.

Link Variables:

HCPID

Next

MANCHESTER 1824

The University of Manchester

Intro

Making Choices About Pharmacogenetic Testing in Primary Care

Thank you for your interest in this study which aims to understand healthcare professionals' opinions on using genetics to guide prescribing in primary care (a concept known as **pharmacogenetics**).

You can take part in this survey even if you have no prior knowledge of pharmacogenetics.

The survey has three parts and will take around 10-15 minutes to finish:

1. Background information on pharmacogenetics (5 minutes)
2. Completing the questionnaire (6 minutes)
3. Questions about you and your views on decision making in healthcare. (2 minutes)

At the end of the study, to compensate you for your time, you have the option of entering a prize draw to win a £25 shopping voucher.

Back Next

0% 100%

MANCHESTER 1824
The University of Manchester

PIS1

Taking part in this research

You are being invited to take part in a research study which aims to understand how genetics can be used in clinical practice to better tailor prescribing. This is a concept known as pharmacogenetics. There is good evidence that this could be used to improve the safety and effectiveness of medicines, but there is no consensus how this might be delivered in the NHS. This study is part of a programme of work to address this.

You will be provided with information about pharmacogenetic testing on the following pages. You do not have to have experience of pharmacogenetic testing to take part in this study.

Before you decide whether to take part in this study it is important for you to understand why the research is being done and what it will involve. The following page contains a summary of key information about the study. For full details about the study please click on the following link to download a copy of the full participant information sheet

https://tinyurl.com/mr3um9as

Back Next

0% 100%

MANCHESTER 1824

The University of Manchester

PIS2

Taking part in this research

Please take time to read the following information carefully. Take time to decide whether or not you wish to take part. Thank you for reading this.

What is the aim of the research?

This study wants to understand what healthcare professionals and members of the public would want from a pharmacogenetic testing service in primary care. The findings will be used to inform the design of a service in England.

Why have I been chosen?

This study aims to explore the views of members of the public and healthcare professionals. Anyone who can read English and has received an email invitation is able to take part in this study.

What happens to the data collected?

The answers to the survey will be used to help us understand how people make decisions about their healthcare preferences and how information can be better provided to help this decision making. Your name will not be recorded and all survey answers will be given an anonymous identification number.

How is confidentiality maintained?

Laws called the Data Protection Act (2018) and General Data Protection Regulation (GDPR) tell us how to keep your information secure. All researchers are trained with this in mind, and your data will be looked after in the following way:

No information which would allow you to be identified will be collected by the research team. Data will be stored in secure University of Manchester servers and computers. The data collected during this study will be stored for 4 years and then destroyed. The data collected in this study will not be shared outside of the members of the research team who are based at the University of Manchester.

Will the outcomes of the research be published?

The main outcome of this research will be a report presenting what members of the public and healthcare professionals perceive as being most important in a pharmacogenetic testing service. In addition, we may want to report the findings at conferences or in a published journal article.

Contact details

If you have any queries about the study then please contact the researcher.

DR JOHN H MCDERMOTT, NIHR DOCTORAL RESEARCH FELLOW IN GENOMIC MEDICINE

Email: john.mcdermott-2@manchester.ac.uk

Telephone: 0161 306 7970

Do you consent to take part in this study?

PIS2=1 Yes

PIS2=2 No

Back Next

Role

Taking part in this research

Which of these options best describes your professional role? *Please only complete this survey if you are a registered healthcare professional.*

Role=1
General Practitioner

Role=2
Physician in Secondary Care (Hospital Based)

Role=3
Pharmacist (Community)

Role=4
Pharmacist (Primary Care)

Role=5
Pharmacist (Secondary Care/Hospital Based)

Role=6 Role_6_other
Other Healthcare Professional

Role=7
I am not a Healthcare Professional

Experience

Have you ever ordered a pharmacogenetic test in the past?

Experience=1
Yes

Experience=2
No

Experience=3
Unsure

whattest

If yes, what pharmacogenetic test have you ordered in the past?

Back Next

0% 100%

MANCHESTER
1824
The University of Manchester

infointro

Thank you for agreeing to take part in this study

We will now show you a video which will provide some background information about pharmacogenetic testing. After you have looked at this information we will ask you some questions about your preferences for pharmacogenetic testing in primary care.

Please press next to continue

Back Next

0% 100%

MANCHESTER 1824

The University of Manchester

sciani

Background Information

Please watch this brief video that explains Pharmacogenetic Testing and its relevance in primary care. The video includes background information and instructions on how to complete the survey.

00:00 / 00:00

Back Next

0% 100%

MANCHESTER 1824

The University of Manchester

quintro

Your preferences for a pharmacogenetic test in primary care

We will now ask you to complete eight questions about your preferences for a pharmacogenetic test in primary care. In each question you will be shown two potential pharmacogenetic tests which vary in different ways. You will be asked to select which pharmacogenetic test you would prefer to offer to a patient. You can also choose not to offer a pharmacogenetic test.

Each test has five characteristics:

1. The ability of the test to improve the effectiveness of a medicine
2. The ability of the test to reduce the risk of adverse drug reactions
3. How you receive the test results
4. The time taken from having the test to receiving the results (turn around time)
5. The type of genetic data returned to the clinical teams

These characteristics and the potential levels they can take are explained over the next five pages

Back Next

0% 100%

MANCHESTER 1824
The University of Manchester

Effectiveness

The Ability of the Test to Improve the Effectiveness of Treatment

One aim of pharmacogenetic guided prescribing is that any results can be used to improve the effectiveness of a given treatment strategy by allowing you to modify the dose or choose alternative medicines. In the hypothetical examples given, the baseline chance of a medicine being effective is 50%. Different types of pharmacogenetic test can increase the effectiveness of a treatment strategy by different amounts. In the scenarios shown to you, there are four levels of effectiveness for you to consider:

**50% (Baseline)**	The pharmacogenetic test does not increase the chance of the subsequent treatment being effective compared to baseline – this means the chance of effectiveness remains at 50%	50%
**60%**	The pharmacogenetic test increases the chance of the treatment strategy being effective from 50% (baseline) to 60%	60%
**70%**	The pharmacogenetic test increases the chance of the treatment strategy being effective from 50% (baseline) to 70%	70%
**80%**	The pharmacogenetic test increases the chance of the treatment strategy being effective from 50% (baseline) to 80%	80%

Back Next

0% 100%

MANCHESTER 1824
The University of Manchester

ADRs

The Ability of the Test to Reduce the Risk of Adverse Drug Reactions

One aim of pharmacogenetic guided prescribing is that any results can be used to improve the safety of a given medicine. In the examples given, without a pharmacogenetic test, the chance of an adverse drug reaction occurring is 20%. Different types of pharmacogenetic test can decrease the risk of an adverse drug reaction by different amounts. In the scenarios shown to you, there are four levels of adverse drug reaction risk for you to consider:

20% (Baseline) The pharmacogenetic test does not reduce the risk of an adverse drug reaction compared to baseline – this means the chance of an adverse drug reaction remains at 20%.

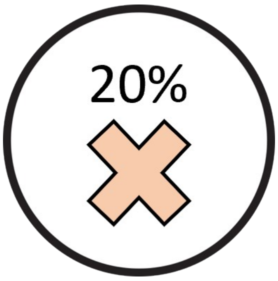

15% The pharmacogenetic test decreases the risk of an adverse drug reaction from 20% (baseline) to 15%.

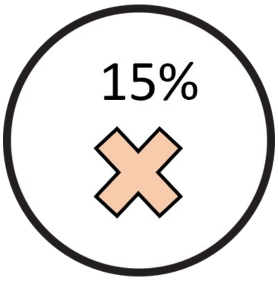

10% The pharmacogenetic test decreases the risk of an adverse drug reaction from 20% (baseline) to 10%.

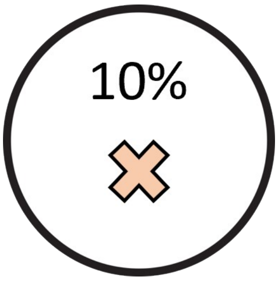

5% The pharmacogenetic test decreases the risk of an adverse drug reaction from 20% (baseline) to 5%.

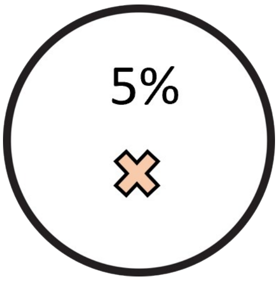

Back Next

0% 100%

MANCHESTER 1824
The University of Manchester

Recieptofresults

How Test Results are Shared With the Clinical Team?

After a sample has been taken, it will be tested in the laboratory and results are made available to healthcare providers. There are four potential approaches for these results to be returned with clinicians:

**Post**	In this scenario, you will receive a letter in the mail with your patient's pharmacogenetic test results.	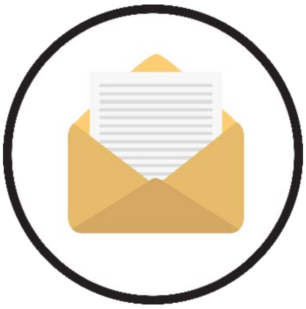
**Email**	In this scenario, you will receive your patient's pharmacogenetic test results via a secure email.	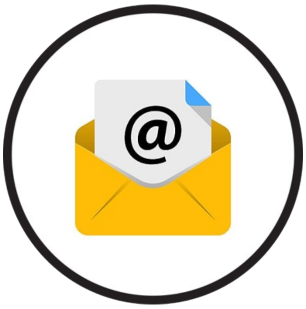
**Web-Portal**	In this scenario, you will be able to view your patient's pharmacogenetic test results via a dedicated website with your own secure login.	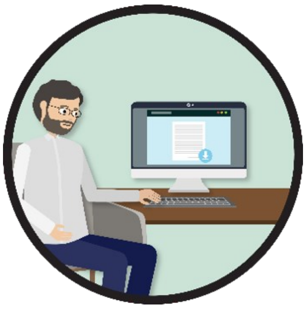
**Within Electronic Health Record**	In this scenario, results will be available within your electronic healthcare record system (i.e. EMIS/SystmOne/EPIC)	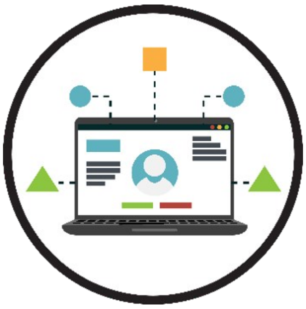

Back Next

0% 100%

MANCHESTER 1824
The University of Manchester

TAT

Time to Result (Turnaround Time)

Once a clinical sample has been taken, it has to be sent to a genetic laboratory for testing. Once the results are returned, you can then use this information to issue a prescription. The time it takes from a patient providing a sample to results being returned is known as the turnaround time or "time to result". In the hypothetical pharmacogenetic tests, there are four possible times to result:

Five (5) Days In this scenario, you will receive the pharmacogenetic results five days after first seeing the patient in clinic.

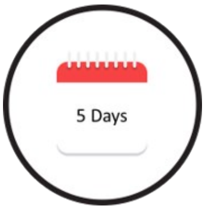

Ten (10) Days In this scenario, you will receive the pharmacogenetic results Ten days after first seeing the patient in clinic.

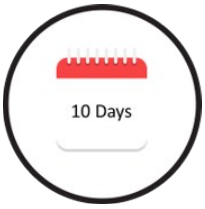

Fifteen (15) Days In this scenario, you will receive the pharmacogenetic results fifteen days after first seeing the patient in clinic.

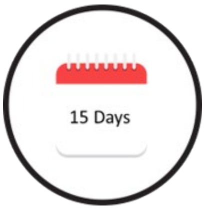

Twenty (20) Days In this scenario, you will receive the pharmacogenetic results twenty days after first seeing the patient in clinic.

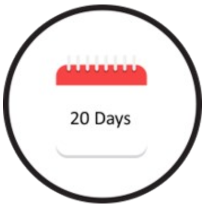

Back Next

0% 100%

MANCHESTER 1824

The University of Manchester

GPResults

The type of genetic data returned to the clinical teams

There are many different types of pharmacogenetic test and many ways in which the data can be reported. This characteristic relates to the wider usefulness of the pharmacogenetic results once they have been returned. In the hypothetical pharmacogenetic tests, there are three approaches to returning results:

Focussed In this scenario, pharmacogenetic results are only provided in relation to a single medicine - i.e. the medicine for which the pharmacogenetic test was initially requested. The results cannot be used to help inform any additional future prescribing activity.

Narrow The pharmacogenetic results will be returned in a format so they can be used to inform future prescribing activity. Prescribing guidance will only be returned where very strong evidence exists for a gene-drug pair. This will provide results for changes across **five** genes which can be used to inform the prescription of approximately **twenty** medicines.

Broad The pharmacogenetic results will be returned in a format so they can be used to inform future prescribing activity. Prescribing guidance will be returned for all gene-drug pairs where there is any evidence, irrespective of strength. This will provide results for changes across **fifty** genes which can be used to inform the prescription of approximately **two hundred** medicines

Back Next

0% 100%

MANCHESTER 1824

The University of Manchester

dceintro1

<u>Completing the Survey</u>

Now that you have seen the potential characteristics of the pharmacogenetic tests, we will show you an example of a choice question.

Below is **<u>an example</u>** where the respondent has been asked to choose between two different pharmacogenetic tests (Test A and Test B), and the option to have no pharmacogenetic test.

They have chosen that they would prefer the second pharmacogenetic test (Test B) and not the first test (Test A) or for there to be no test. They have made this choice by clicking the "select" button at the bottom of each option.

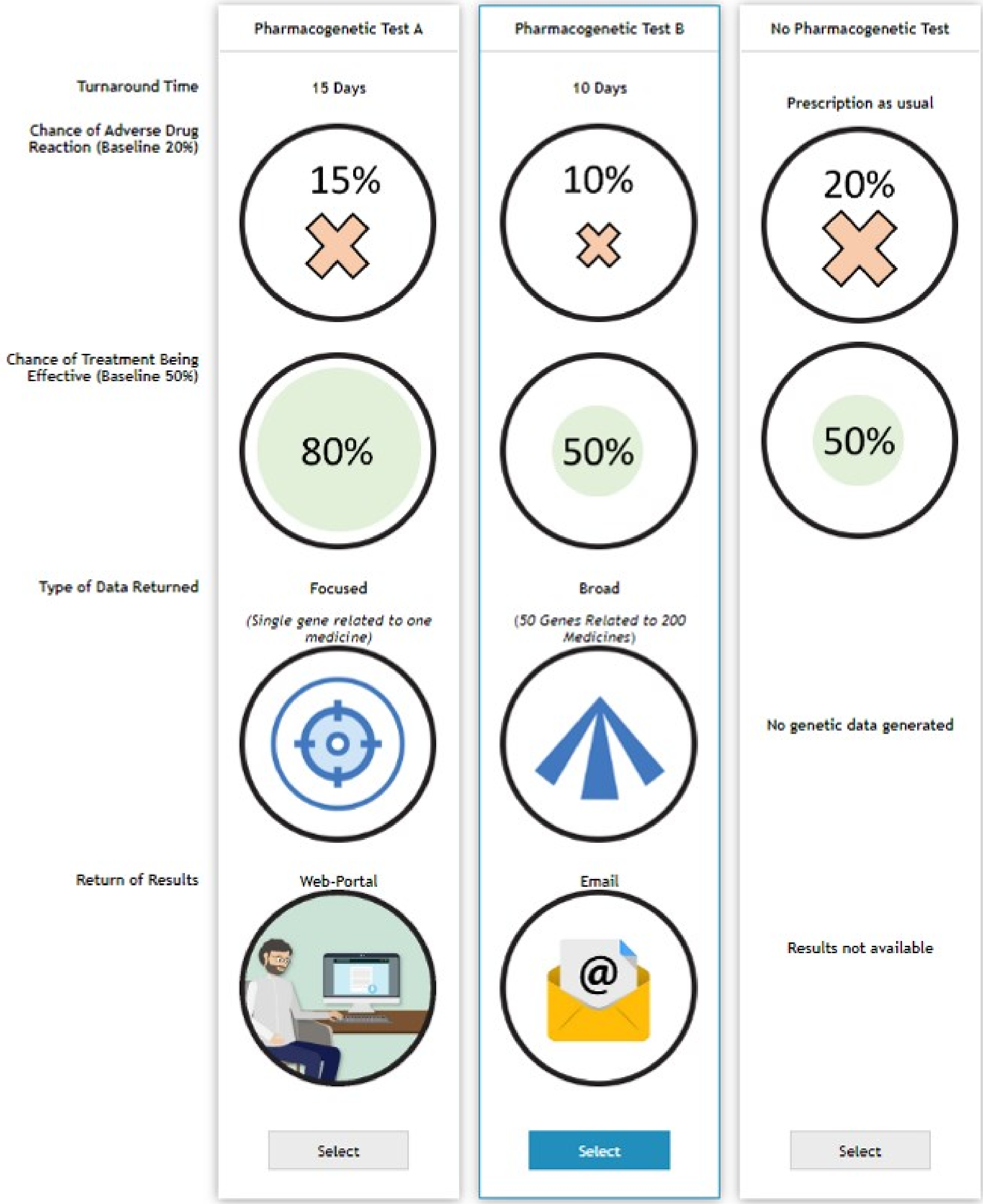

We will now ask you to complete eight questions. Depending on the screen you are using to complete this survey, you may need to scroll down to view all the attributes within each test and access the "select" button.

Back Next

The University of Manchester

IntroDep

Imagine A Patient Presents to You With Low Mood

When considering your answers, imagine a patient presents to you with low mood which they have had for several months. As well as referring the patient for psychological support, you consider a trial of an antidepressant.

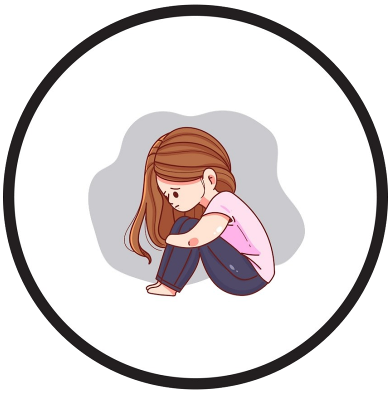

To help guide your treatment plan there is a **new genetic test which can be used to help identify the most suitable medicine for your patient**; increasing the chance that the medicine will work and lowering the risk side effects. This is known as a **pharmacogenetic test**.

You will be asked to choose from two different testing options (Test A or Test B) to offer your patient. You also have the option to not offer the test (No pharmacogenetic test)

0">

skipdep

Back Next

0% 100%

MANCHESTER 1824
The University of Manchester

landdep

DCEDepressionBlock1_Fixed1

Question 1 of 8: If you had to choose one of these pharmacogenetic (PGx) tests (Test A or Test B) to help guide your treatment, which would you choose? *When considering your answers, you should imagine a patient has presented to you with low mood and you are ordering a pharmacogenetic test to help guide antidepressant therapy.*

	Pharmacogenetic Test A	Pharmacogenetic Test B	No Pharmacogenetic Test
Time to Result (Turnaround Time)	15 Days	10 Days	Prescription as usual
Chance of Adverse Drug Reaction (Baseline 20%)	15%	10%	20%
Chance of Treatment Being Effective (Baseline 50%)	80%	50%	50%
Type of Data Returned	Focused<br>*(Single gene related to one medicine)*<br>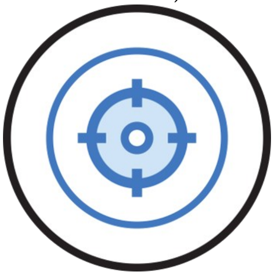	Broad<br>*(50 Genes Related to 200 Medicines)*<br>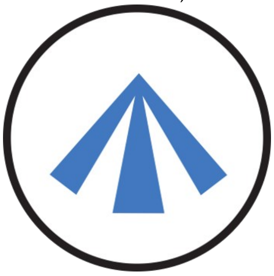	No genetic data generated
Return of Results	Web-Portal<br>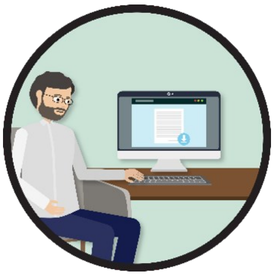	Email<br>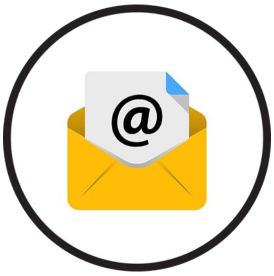	Results not available

DCEDepressionBlock1_Fixed1

Select

DCEDepressionBlock1_Fixed1

Select

DCEDepressionBlock1_Fixed1

Select

Back Next

0% 100%

MANCHESTER 1824

The University of Manchester

DCEDepressionBlock1_Fixed3

Question 3 of 8: If you had to choose one of these pharmacogenetic (PGx) tests (Test A or Test B) to help guide your treatment, which would you choose? *When considering your answers, you should imagine a patient has presented to you with low mood and you are ordering a pharmacogenetic test to help guide antidepressant therapy.*

	Pharmacogenetic Test A	Pharmacogenetic Test B	No Pharmacogenetic Test
Time to Result (Turnaround Time)	15 Days	10 Days	Prescription as usual
Chance of Adverse Drug Reaction (Baseline 20%)	20%	5%	20%
Chance of Treatment Being Effective (Baseline 50%)	80%	50%	50%
Type of Data Returned	Broad *(50 Genes Related to 200 Medicines)*	Narrow *(5 Genes Related to 20 Medicines)*	No genetic data generated
Return of Results	Results Via Post	Email	Results not available
	DCEDepressionBlock1_Fixed3	DCEDepressionBlock1_Fixed3	DCEDepressionBlock1_Fixed3

Select

Select

Select

Back

Next

0% 100%

MANCHESTER 1824

The University of Manchester

DCEDepressionBlock1_Fixed4

Question 4 of 8: If you had to choose one of these pharmacogenetic (PGx) tests (Test A or Test B) to help guide your treatment, which would you choose? *When considering your answers, you should imagine a patient has presented to you with low mood and you are ordering a pharmacogenetic test to help guide antidepressant therapy.*

	Pharmacogenetic Test A	Pharmacogenetic Test B	No Pharmacogenetic Test
Time to Result (Turnaround Time)	5 Days	20 Days	Prescription as usual
Chance of Adverse Drug Reaction (Baseline 20%)	20%	5%	20%
Chance of Treatment Being Effective (Baseline 50%)	60%	70%	50%
Type of Data Returned	Broad<br>*(50 Genes Related to 200 Medicines)*<br>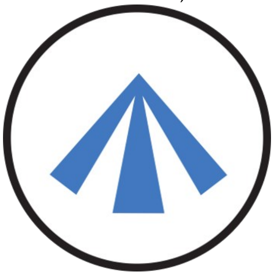	Narrow<br>*(5 Genes Related to 20 Medicines)*<br>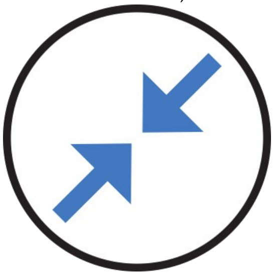	No genetic data generated
Return of Results	Email<br>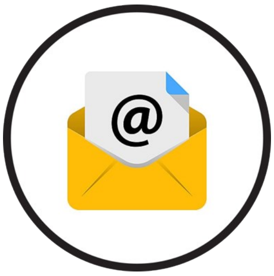	Web-Portal<br>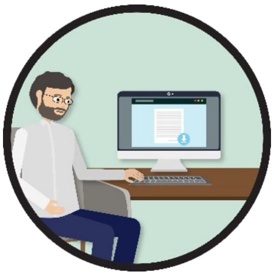	Results not available

DCEDepressionBlock1_Fixed4 DCEDepressionBlock1_Fixed4 DCEDepressionBlock1_Fixed4

Select

Select

Select

Back Next

0% 100%

MANCHESTER 1824

The University of Manchester

DCEDepressionBlock1_Fixed5

Question 5 of 8: If you had to choose one of these pharmacogenetic (PGx) tests (Test A or Test B) to help guide your treatment, which would you choose? *When considering your answers, you should imagine a patient has presented to you with low mood and you are ordering a pharmacogenetic test to help guide antidepressant therapy.*

	Pharmacogenetic Test A	Pharmacogenetic Test B	No Pharmacogenetic Test
Time to Result (Turnaround Time)	10 Days	15 Days	Prescription as usual
Chance of Adverse Drug Reaction (Baseline 20%)	10%	15%	20%
Chance of Treatment Being Effective (Baseline 50%)	60%	70%	50%
Type of Data Returned	Focused<br>*(Single gene related to one medicine)*	Narrow<br>*(5 Genes Related to 20 Medicines)*	No genetic data generated
Return of Results	Email	Embedded into EHR	Results not available
	DCEDepressionBlock1_Fixed5	DCEDepressionBlock1_Fixed5	DCEDepressionBlock1_Fixed5

Select

Select

Select

Back

Next

0% 100%

MANCHESTER 1824

The University of Manchester

DCEDepressionBlock1_Fixed6

Question 6 of 8: If you had to choose one of these pharmacogenetic (PGx) tests (Test A or Test B) to help guide your treatment, which would you choose? *When considering your answers, you should imagine a patient has presented to you with low mood and you are ordering a pharmacogenetic test to help guide antidepressant therapy.*

	Pharmacogenetic Test A	Pharmacogenetic Test B	No Pharmacogenetic Test
Time to Result (Turnaround Time)	5 Days	20 Days	Prescription as usual
Chance of Adverse Drug Reaction (Baseline 20%)	10%	15%	20%
Chance of Treatment Being Effective (Baseline 50%)	70%	60%	50%
Type of Data Returned	Broad<br>*(50 Genes Related to 200 Medicines)*<br>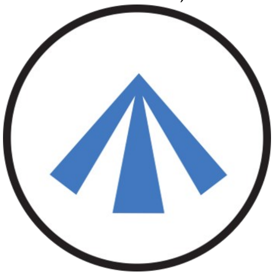	Focused<br>*(Single gene related to one medicine)*<br>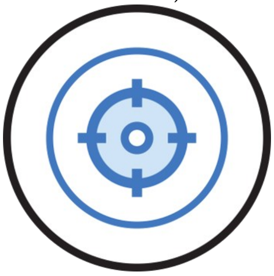	No genetic data generated
Return of Results	Embedded into EHR<br>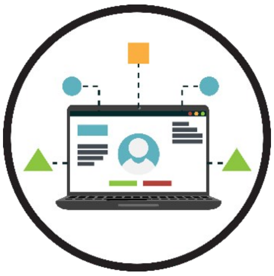	Results Via Post<br>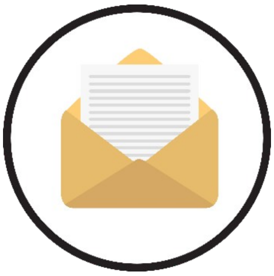	Results not available

DCEDepressionBlock1_Fixed6 DCEDepressionBlock1_Fixed6 DCEDepressionBlock1_Fixed6

Select Select Select

Back Next

0% 100%

MANCHESTER 1824

The University of Manchester

DCEDepressionBlock1_Fixed7

Question 7 of 8: If you had to choose one of these pharmacogenetic (PGx) tests (Test A or Test B) to help guide your treatment, which would you choose? *When considering your answers, you should imagine a patient has presented to you with low mood and you are ordering a pharmacogenetic test to help guide antidepressant therapy.*

	Pharmacogenetic Test A	Pharmacogenetic Test B	No Pharmacogenetic Test
Time to Result (Turnaround Time)	20 Days	5 Days	Prescription as usual
Chance of Adverse Drug Reaction (Baseline 20%)	15%	10%	20%
Chance of Treatment Being Effective (Baseline 50%)	50%	80%	50%
Type of Data Returned	Focused<br>*(Single gene related to one medicine)*<br>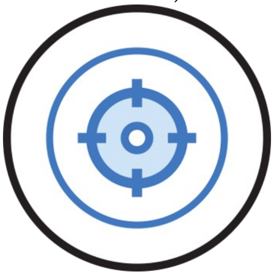	Narrow<br>*(5 Genes Related to 20 Medicines)*<br>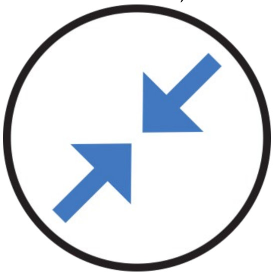	No genetic data generated
Return of Results	Web-Portal<br>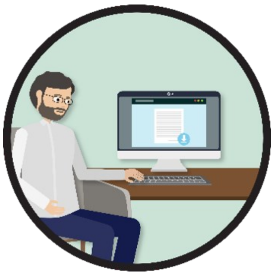	Embedded into EHR<br>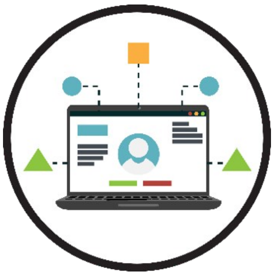	Results not available

DCEDepressionBlock1_Fixed7 DCEDepressionBlock1_Fixed7 DCEDepressionBlock1_Fixed7

Select Select Select

Back Next

0% 100%

MANCHESTER 1824

The University of Manchester

DCEDepressionBlock1_Fixed8

Question 8 of 8: If you had to choose one of these pharmacogenetic (PGx) tests (Test A or Test B) to help guide your treatment, which would you choose? *When considering your answers, you should imagine a patient has presented to you with low mood and you are ordering a pharmacogenetic test to help guide antidepressant therapy.*

	Pharmacogenetic Test A	Pharmacogenetic Test B	No Pharmacogenetic Test
Time to Result (Turnaround Time)	20 Days	5 Days	Prescription as usual
Chance of Adverse Drug Reaction (Baseline 20%)	5%	20%	20%
Chance of Treatment Being Effective (Baseline 50%)	70%	60%	50%
Type of Data Returned	Focused<br>*(Single gene related to one medicine)*<br>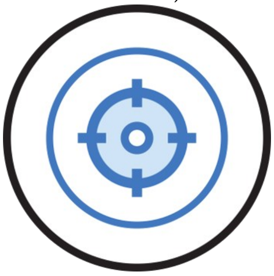	Narrow<br>*(5 Genes Related to 20 Medicines)*<br>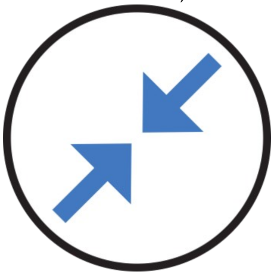	No genetic data generated
Return of Results	Embedded into EHR<br>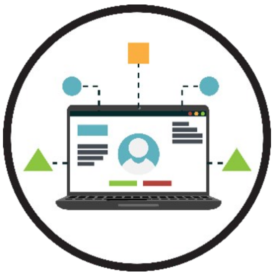	Web-Portal<br>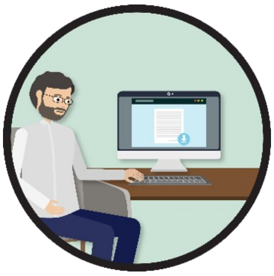	Results not available

DCEDepressionBlock1_Fixed8 DCEDepressionBlock1_Fixed8 DCEDepressionBlock1_Fixed8

Select

Select

Select

Back

Next

0% 100%

The University of Manchester

skip4

Back Next

0% 100%

MANCHESTER 1824

The University of Manchester

check

<u>We would now like to ask you some questions about how you found answering these questions</u>

Q9: How confident are you that you would make the same choices if faced with the situations in real-life?

check=1 Very confident I would make the same choices

check=2 Quite confident I would make the same choices

check=3 Not confident I would make the same choices

difficulty

Q10: On a scale of 1 to 5, how easy or difficult did you find making choices between the alternatives?

difficulty=1 1 Very easy

difficulty=2 2 Quite easy

difficulty=3 3 Neither easy or difficult

difficulty=4 4 Quite difficult

difficulty=5 5 Very difficult

Back Next

0% 100%

MANCHESTER 1824

The University of Manchester

nonattendance

Q11: Did you find yourself making choices based on the same attribute each time? *(i.e. did you think one attribute was more important to you than others)*

nonattendance=1 Yes, I focussed on one of the attributes more than others

nonattendance=2 No, I used all of the attributes to make my choices

Back Next

0% 100%

MANCHESTER 1824

The University of Manchester

attributechoice

Q12: Which attributes did you use to make your choices (you can select more than one)?

attributechoice_1 ☐ Effectiveness

attributechoice_2 ☐ Risk of adverse drug reactions

attributechoice_3 ☐ Time to Result (Turnaround Time)

attributechoice_4 ☐ How you receive the results

attributechoice_5 ☐ The type of data reported

Back Next

0% 100%

MANCHESTER 1824

The University of Manchester

GeneticsDecisions

Q13. Please indicate which of the following statements best reflects your views of using genetics in healthcare.

GeneticsDecisions=1 I think that genetics could be used to improve patient outcomes across all areas of healthcare

GeneticsDecisions=2 I think that genetics has the potential to improve patient outcomes, but only in certain areas of healthcare

GeneticsDecisions=3 I think that genetics is useful to investigate rare disease and cancer, but is not that useful in other areas of medicine

GeneticsDecisions=4 I think that genetics isn't useful in healthcare

GeneticsDecisions=5 No Opinion

Back Next

0% 100%

MANCHESTER 1824

The University of Manchester

Agenumber

We would now like to ask you some questions about yourself

Q14: How old are you (in years)? ☐

Gender

Q15: How would you describe your gender identity?

- Gender=1 ○ Male
- Gender=2 ○ Female
- Gender=3 ○ Non-Binary
- Gender=4 ○ Prefer not to answer

Ethnicity

Q16: Which race or ethnicity best describes you? (Please choose only one.)

- Ethnicity=1 ○ English, Welsh, Scottish, Northern Irish or British
- Ethnicity=2 ○ Irish
- Ethnicity=3 ○ Gypsy or Irish Traveller
- Ethnicity=4 ○ Roma
- Ethnicity=5 ○ Any other White background
- Ethnicity=6 ○ Indian
- Ethnicity=7 ○ Pakistani
- Ethnicity=8 ○ Bangladeshi
- Ethnicity=9 ○ Chinese
- Ethnicity=10 ○ Any other Asian background
- Ethnicity=11 ○ Caribbean
- Ethnicity=12 ○ African

Ethnicity=13
Any other Black, Black British, or Caribbean background

Ethnicity=14
Arab

Ethnicity=15
Mixed Ethnicity: White and Black Caribbean

Ethnicity=16
Mixed Ethnicity: White and Black African

Ethnicity=17
Mixed Ethnicity: White and Asian

Ethnicity=18
Mixed Ethnicity: Any other Mixed or multiple ethnic background

Ethnicity=19
Ethnicity_19_other
Other (please specify)

dephistory

Q17: Have you ever been prescribed antidepressant medicines by your doctor?

dephistory=1
Yes

dephistory=2
No

dephistory=3
Unsure

dephistory=4
Decline to answer

jphistory

Q18: Have you ever been prescribed medicine for pain relief by your doctor?

jphistory=1
Yes

jphistory=2
No

jphistory=3
Unsure

jphistory=4
Decline to answer

yourmeds

Q19: Do you take regular medicines?

yourmeds=1
Yes

yourmeds=2
No

yourmeds=3
Unsure

yourmeds=4 Decline to answer

IssueswithSE

Q20. Have you personally had side effects from medicines in the past?

IssueswithSE=1 Yes

IssueswithSE=2 No

IssueswithSE=3 Unsure

IssueswithSE=4 Decline to answer

Back Next

0% 100%

The University of Manchester

comments

Q21: Do you have any comments or feedback on this survey?

Back Next

0% 100%

MANCHESTER 1824

The University of Manchester

email

Remuneration - Prize Draw

Many thanks for taking part in this research. For taking part, you are eligible to enter a prize draw to win a £25 gift voucher. There are 40 vouchers to be won in total and winners will be notified by email once the study has closed.

To enter the prize draw you will need to provide your email. This will be stored on a secure server at the University of Manchester and will not be used for any purposes other than for the draw. Once the draw is complete, all emails will be permanently deleted.

Emaildetail

If you wish to enter the prize draw, please enter your email. Otherwise, leave blank. *Please note, this should be a professional email address which will be used for validation.*

Back Next

0% 100%

MANCHESTER 1824

The University of Manchester

finish1

Thank you for taking part in this study

Back Next

0% 100%

MANCHESTER 1824

The University of Manchester

finish

Thank you for taking part in this study. You may now close your browser.

0% 100%

MANCHESTER 1824
The University of Manchester

disqualify

Thank you for your interest in this survey. Based on your answers, this survey is not suitable for you. You may now close your browser.

Note:

When respondents take the survey in regular mode this page will not be displayed. Respondents will be redirected to the url below:

0% 100%

MANCHESTER 1824

The University of Manchester

endquota

Note:

When respondents take the survey in regular mode this page will not be displayed.
Respondents will be redirected to the url below:

0% 100%

MANCHESTER 1824
The University of Manchester
